# Comparison of two area-level socioeconomic deprivation indices: Implications for public health research, practice, and policy

**DOI:** 10.1371/journal.pone.0292281

**Published:** 2023-10-05

**Authors:** Kimberly A. Rollings, Grace A. Noppert, Jennifer J. Griggs, Robert A. Melendez, Philippa J. Clarke

**Affiliations:** 1 Institute for Healthcare Policy and Innovation, Health & Design Research Fellowship Program, University of Michigan, Ann Arbor, Michigan, United States of America; 2 Institute for Social Research, Social Environment and Health, Survey Research Center, University of Michigan, Ann Arbor, Michigan, United States of America; 3 Department of Medicine, Division of Hematology/Oncology, University of Michigan, Ann Arbor, Michigan, United States of America; 4 Department of Health Management and Policy, School of Public Health, University of Michigan, Ann Arbor, Michigan, United States of America; 5 Institute for Healthcare Policy and Innovation, University of Michigan, Ann Arbor, Michigan, United States of America; Wuhan University, CHINA

## Abstract

**Objectives:**

To compare 2 frequently used area-level socioeconomic deprivation indices: the Area Deprivation Index (ADI) and the Social Vulnerability Index (SVI).

**Methods:**

Index agreement was assessed via pairwise correlations, decile score distribution and mean comparisons, and mapping. The 2019 ADI and 2018 SVI indices at the U.S. census tract-level were analyzed.

**Results:**

Index correlation was modest (R = 0.51). Less than half (44.4%) of all tracts had good index agreement (0–1 decile difference). Among the 6.3% of tracts with poor index agreement (≥6 decile difference), nearly 1 in 5 were classified by high SVI and low ADI scores. Index items driving poor agreement, such as high rents, mortgages, and home values in urban areas with characteristics indicative of socioeconomic deprivation, were also identified.

**Conclusions:**

Differences in index dimensions and agreement indicated that ADI and SVI are not interchangeable measures of socioeconomic deprivation at the tract level. Careful consideration is necessary when selecting an area-level socioeconomic deprivation measure that appropriately defines deprivation relative to the context in which it will be used. How deprivation is operationalized affects interpretation by researchers as well as public health practitioners and policymakers making decisions about resource allocation and working to address health equity.

## Introduction

Numerous studies document associations between area-level socioeconomic deprivation and poor health outcomes, including reduced life expectancy, higher morbidity, and reduced access to preventive healthcare [1–4]. Studies often rely on publicly available composite measures that include multiple socioeconomic dimensions such as income, employment, education, and housing characteristics [5]. Such indices are used in population-based studies, public health interventions, and policy decisions [3], including healthcare payment adjustments by social risk [3,6] and COVID-19 vaccination allocation and planning [7,8]. However, understanding index creation, content, scoring, and spatial scale is critical for index selection by researchers, public health practitioners, and policymakers, especially when index use informs resource allocation decisions and efforts to address health equity.

Despite prior work examining substantive index differences, detailed quantitative differences between indices commonly used in a public health context are still unclear. Prior studies described similarities and differences in index development, domains, items, scoring, data sources, scale, availability, and intended use [1,3,6,7,9]. Other work has largely focused on index differences at varying geographic scales [5,10,11]. Although there is no consensus on which area-level socioeconomic deprivation measure to use, consideration of index differences is necessary to select an appropriate measure.

The present study compared two frequently used area-level socioeconomic deprivation indices: the Area Deprivation Index (ADI; University of Wisconsin) and the Social Vulnerability Index (SVI; Centers for Disease Control and Prevention, CDC). These indices are the most widely adopted indices in public health and health services research, practice, and policy-making, yet indices are often used without much consideration of their differences or implications [6,8,10,12,13]. Based on existing literature and publicly available index data, the present study compared ADI and SVI to quantify and map index differences at the census tract level across the U.S. A more detailed understanding of index differences is necessary for evaluation of existing and proposed research, funding and resource allocation, and policy using these measures. This research is timely considering increased interest in the health impacts of neighborhood and socioeconomic contexts, social determinants of health, health equity, and recommended immediate use of ADI and SVI for national policy development addressing health-related social needs [6,13].

## Methods

### Area-level socioeconomic deprivation indices

#### Area Deprivation Index (ADI)

The ADI assesses neighborhood deprivation by census block group (600–3000 people). Developed by Singh in 2003 [2] and adapted by Kind and colleagues in 2014 [14,15], the factor-based index includes 17 U.S. census indicators of income, education, employment, and housing quality (Table 1) [2]. Each indicator is multiplied by its factor score coefficient and then summed within each block group. Poverty, income, and education are weighted most heavily among the 17 items. The resulting sums are then converted into a standardized index via arbitrarily setting the index mean at 100 and standard deviation at 20 [2,14]. The ADI national percentile rankings of block groups range from 0 to 100, with 100 being the greatest level of deprivation [2]. Decile rankings are also available for individual states. The ADI is not released as raw scores and instructs users to apply ADI in rank-type format only due to its construction [16].

**Table 1:** Comparison of ADI and SVI items.

Domain	Item^a^	Index (# items)	
		ADI (17)	SVI (15)
Income	Below poverty level	•	•
	Below 150% of poverty level	•	
	Income disparity	•	
	Median family income	•^b^	
	Per capita income		•
Employment	Unemployment	•	•
	White collar occupation	•^b^	
Education	High school diploma or higher	•^b^	
	≤ High school diploma		•
	<9 years of education	•	
Housing	Owner-occupied housing	•^b^ ^c^	
	Median monthly mortgage	•^b^ ^c^	
	Median gross rent	•^b^	
	Median home value	•^b^ ^c^	
Household	Single-parent households	•	•
Characteristics	Age 65+ years (older adults)		•
	Age ≤17 years (children)		•
	Persons with a disability		•
	Households w/out a telephone	•	
	Households w/out a motor vehicle	•^c^	•
	Housing w/out complete plumbing	•^c^	
Housing Type	Multi-unit (10+) structures		•
	Crowding (>1 person/room)	•^c^	•
	Mobile homes		•
	Persons in group quarters		•
Minority Status and Language	Non-Hispanic White		•
	Speak English “less than well”		•

*Abbreviations*: ADI, area deprivation index; SVI, social vulnerability index.

^a^ = For complete ADI and SVI item information, see S2 Table.

^b^ = Indicates negative factor loadings.

^c^ = Tract factor loadings below 0.65.[2].

ADI 2019 data, based on 2015–2019 American Community Survey (ACS) data, were downloaded from the Neighborhood Atlas website [16]. ADI was calculated for block groups in the 50 U.S. states, DC, and Puerto Rico, excluding those with less than 30 housing units, 100 people, or more than one-third of the population residing in group quarters [17]. Block groups with known errors acknowledged by the Census Bureau were also excluded [16]. To facilitate index comparisons, the ADI was aggregated from block group to census tract using population-weighted means to match the geographic scale of the SVI. Census-tract level scores were then ranked by decile to enable direct comparisons.

#### Social Vulnerability Index (SVI)

The SVI was developed to spatially identify vulnerable communities most likely requiring support when preparing for, responding to, and recovering from natural disasters, hazardous events, and disease outbreaks [7,18,19]. SVI (2018) measures social vulnerability at the tract level (4,000 people, on average) based on 15 indicators within four themes: socioeconomic status (SES), household composition and disability, minority status and language, and housing type and transportation (Table 1). Socially vulnerable populations include people with disabilities, older adults, children, people without vehicles, and people with limited English proficiency. Percentile rankings of all 15 items are calculated for individual items, the four themes, and a geographic unit’s overall ranking. Individual item percentiles are summed for each theme and within each geographic unit. Summed percentiles are then ordered to identify theme-specific percentile rankings. For each of the 4 themes, percentiles of individual items within each theme are summed. SVI’s relative rankings based on percentiles are available nationally, by state, by county, and by census tract [18]. SVI data based on 2014–2018 ACS data were downloaded from the CDC SVI website [18].

#### ADI and SVI characteristics

ADI and SVI share data sources but vary by index purpose, domains, items, construction, scoring method, and availability (frequency and geographic scale) [3,6,7,12]. ADI quantifies neighborhood (block group) level deprivation via variations in SES [16], while SVI aims to identify under-resourced areas requiring support before, during, and after hazardous or emergency events [18,19]. Each index contains unique domains and items that define deprivation differently. Detailed index and index item descriptions are available in S1 and S2 Tables.

Table 1 illustrates the 7 author-identified domains and 27 unique items addressed by the indices. ADI included 17 items across 6 domains: income, employment, education, housing, household characteristics, and housing type. SVI included 15 items across 6 domains: income, employment, education, household characteristics, housing type, and minority status and language. Indices shared 5 items in common across 4 domains: below poverty level (income), unemployment (employment), single-parent households and households without a motor vehicle (household characteristics), and crowded households (housing type). ADI contained unique items within the domains of income (below 150% of the poverty level, median family income, and income disparity), employment (white collar occupations), education (high school education or higher, less than 9 years of education), housing (owner-occupied housing, median monthly mortgage, median gross rent, median home value), and household characteristics (without a telephone, without complete plumbing). SVI contained unique items within the domains of income (per capita income), education (less than a high school diploma), household (age 65 years and older, under 17 years of age, disability status), housing type (multi-unit structures, mobile homes, group quarters), and minority status and language.

### Analysis

Index agreement was assessed in three ways: pairwise Spearman correlations, decile score distribution and mean index score comparisons, and mapping. Spearman correlation coefficients were first generated to examine overall associations between continuous index scores (nationwide rankings), as well as between scores categorized by decile (“decile scores”). Next, ADI and SVI decile score distributions were reviewed to define “good” and “poor” index agreement. Considering that ADI and SVI are often used to identify areas with high deprivation, such as the top decile, quartile, or quintile [13,20–22], subsequent analyses focused on index agreement between tracts with the highest and lowest deprivation decile scores. Good index agreement was defined by a 0 to 1 decile difference between ADI and SVI scores for each tract. Poor index agreement was defined by a difference of at least 6 deciles between ADI and SVI scores. The Results section describes in detail how the distribution of decile scores informed these definitions. Third, mean ADI and SVI scores for tracts with good and poor index agreement, within the 10% highest and 10% lowest deprivation deciles, were compared to quantify “mismatched” tracts among tracts with the highest and lowest deprivation decile scores.

ADI and SVI items driving poor index agreement at the tract level were then identified via t-tests and mapping. T-tests compared individual index item means for tracts with good and poor index agreement. Cohen’s D [classified according to three thresholds: small (|≥0.20 to <0.50|), medium (|≥0.50 to <0.80|), and large (|≥0.80|) effect sizes] was calculated to assess effect sizes of the item mean differences and identify tract characteristics driving poor index agreement [23]. Tracts were mapped by index agreement using Geographic Information Systems (GIS) software and geospatial data [24] to explore geospatial patterns in poor index agreement. All analyses were completed using STATA statistical software version 17 (STATACorp) and ArcGIS Pro version 2.9.5 (ESRI).

## Results

### Index agreement: Correlations

ADI and SVI scores were available for 71,724 of 74,001 (96.9%) tracts due to missing or suppressed census tract data [16]. S3 Table contains ADI and SVI composite scores, individual item means, and descriptive statistics. Correlation coefficients were 0.51 for both comparisons between ADI and SVI original continuous and decile data scores. The lack of change in correlation coefficients for continuous and decile scores suggested that subsequent index comparisons were not confounded by the use of decile versus continuous index data.

### Index agreement: Decile distribution

**Fig 1:**
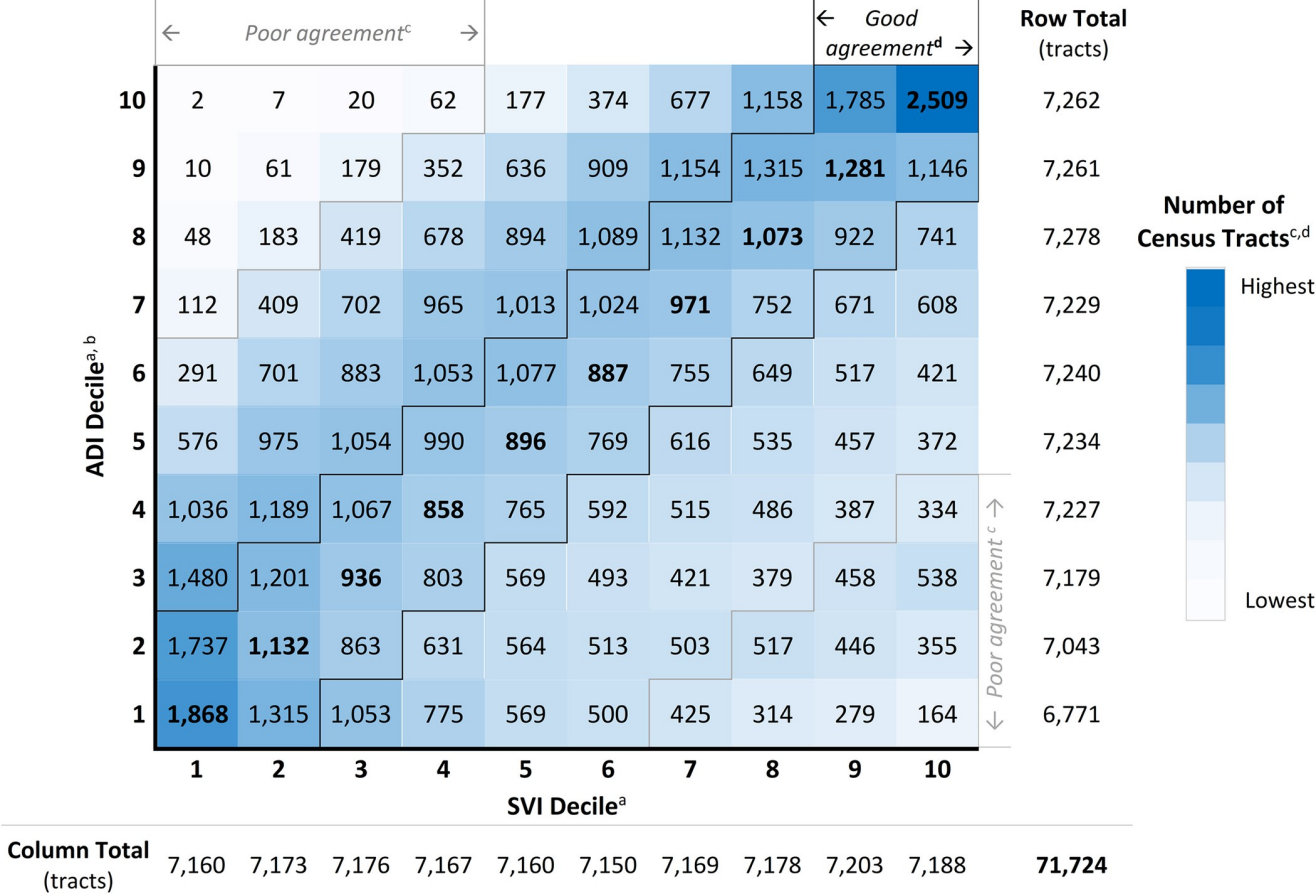
Distribution of census tracts classified by ADI and SVI scores. *Abbreviations*: ADI, area deprivation index; SVI, social vulnerability index. ^a^ = For each index, scores were categorized by decile. ^b^ = For this study, a population-weighted mean was used to aggregate ADI block group data to tract. ^c^ = Census tracts within light lines (upper left and lower right corners) had “poor agreement,” or index scores that differed by at least 6 deciles [6.3% (4,514 tracts)]. Good and poor index agreement tracts within the highest (10) and lowest (1) index score deciles were analyzed in this study (Figs 1 and 3). ^d^ = Tract counts within dark lines had “good agreement,” or index scores that differed by 0 to 1 decile [44.4% (31,829 tracts)]. **Bold text** within the dark lines indicates census tracts with ADI and SVI scores within the same decile.

Of the 71,724 total tracts with available ADI and SVI data, 44.4% had good index agreement. Fig 1 displays the distribution of census tracts across ADI and SVI decile scores. Cell shading darkens as tract counts increase. Values within cells outlined with dark lines indicate tract counts with “good” index agreement (ADI and SVI scores within 0 to 1 decile). The 6.3% of 71,724 census tracts within upper left and lower right corners of Fig 1, outlined with light lines, had “poor” index agreement. A 6-decile or more difference between ADI and SVI scores was used to define poor index agreement based on irregularities in the ADI-SVI decile score distribution (Fig 1). The increasing and decreasing tract counts in the bottom rows of SVI decile columns 7, 8, 9, and 10 were unusual for two positively correlated measures.

**Fig 2:**
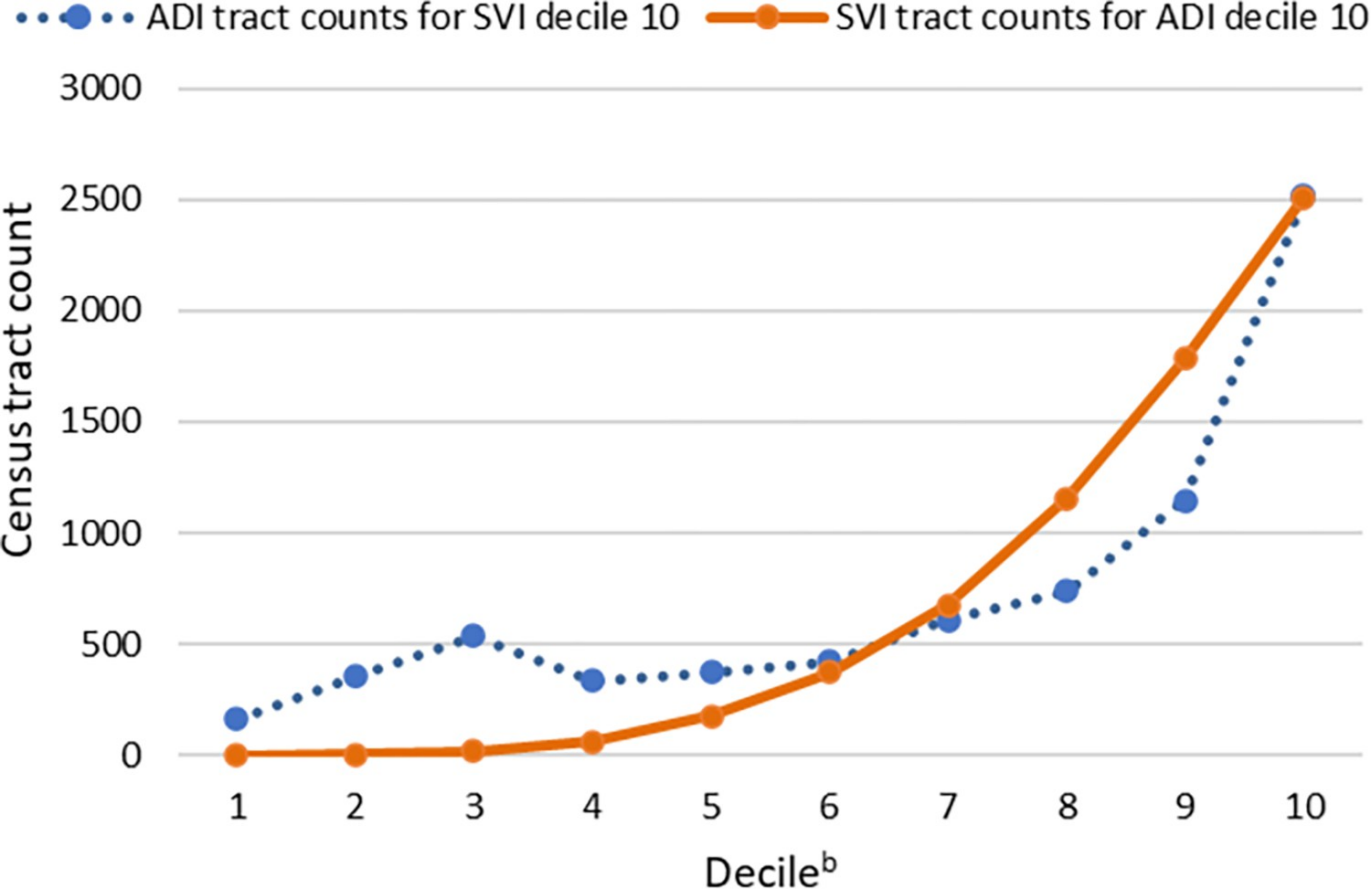
Distribution of census tracts classified by high ADI^a^ and SVI decile scores. *Abbreviations*: ADI, area deprivation index; SVI, social vulnerability index. ^a^ = For this study, a population-weighted mean was used to aggregate ADI block group data to tract. ^b^ = For each index, scores were categorized by decile. High scores indicate high deprivation.

To further illustrate this irregularity, Fig 2 displays tract counts from the highest ADI (Fig 1, top row) and SVI (Fig 1, far right column) deciles. The increase and decrease between deciles 3 and 4, a trend also present in similar comparisons for deciles 7, 8, and 9, determined the definition of poor index agreement.

### Index agreement: Mean index score comparisons

**Fig 3:**
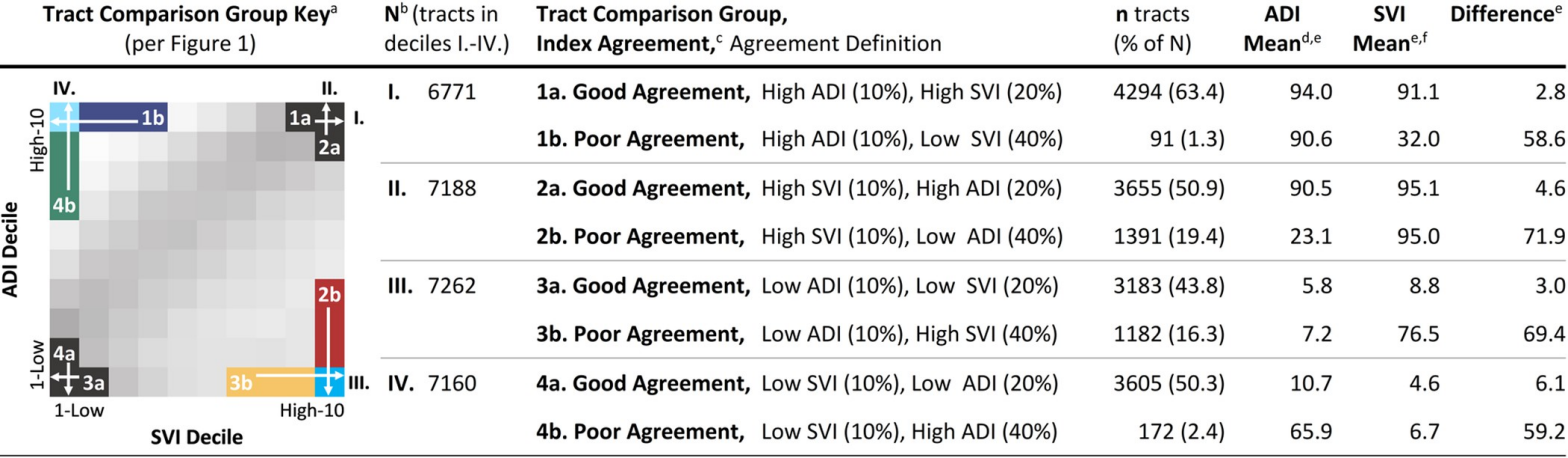
Comparisons of mean ADI and SVI scores among tracts with good and poor index agreement. *Abbreviations*: ADI, area deprivation index; SVI, social vulnerability index. ^a^ = The image illustrates the 8 census tract comparison groups with respect to the distribution of tracts by ADI and SVI decile scores shown in Fig 1. ^b^ = N indicates the total number of tracts within a comparison decile, the first decile noted in the agreement definition [e.g., I. 6,771 total tracts in the top ADI (10%) decile]. ^c^ = Good index agreement was defined as index scores that differed by 0 to 1 decile. Poor index agreement was defined as index scores that differed by at least 6 deciles. ^d^ = A population-weighted mean was used to aggregate ADI block group data to tract. ADI is a percentile ranking from 0 to 100. ^e^ = Higher mean index scores indicated higher deprivation levels. ^f^ = Mean SVI scores (percentile rankings from 0 to 1) were multiplied by 100 for comparison to ADI scores.

Fig 3 contains 8 mean ADI and SVI comparisons (4 groups) of good and poor agreement tracts.

#### Comparison I

Comparison I. identified 6,771 tracts with the highest 10% ADI deprivation scores and then compared mean ADI and SVI scores within good [1a. high ADI (10%), high SVI (20%)] and poor [1b. high ADI (10%), low SVI (40%)] agreement tracts. Mean ADI (94.0) and SVI (91.1) differed by 2.8 among 4,294 good agreement tracts (1a. 63.4% of 6771 tracts). Mean ADI (90.6) and SVI (32.0) differed by 58.6 for 91 poor index agreement tracts (1b. 1.3% of 6771 tracts).

#### Comparison II

Comparison II. assessed tracts with the highest 10% SVI deprivation scores. Of those 7,188 tracts, 50.9% (3655 tracts) had good index agreement [2a. high SVI (10%), high ADI (20%)] with a mean ADI (90.5)-SVI (95.1) difference of 4.6. Mean ADI (23.1) and SVI (95.0) differed by 71.9 for the 19.4% (1391) of poor index agreement tracts [2b. high SVI (10%), low ADI (40%)].

#### Comparison III

Comparison III. selected tracts with the lowest 10% ADI deprivation scores. Of those 7,262 tracts, 43.8% (3183 tracts) had good index agreement [3a. low ADI (10%), low SVI (20%)] with a mean ADI (5.8)-SVI (8.8) difference of 3.0. Mean ADI (7.2) and SVI (76.5) differed by 69.4 among the 16.3% (1182) of poor index agreement tracts [3b. low ADI (10%), high SVI (40%)].

#### Comparison IV

Comparison IV. identified tracts with the lowest 10% SVI deprivation scores. Of those 7,160 tracts, 50.3% (3605 tracts) had good index agreement [4a. low SVI (10%), low ADI (20%)] with a mean ADI (10.7)-SVI (4.6) difference of 6.1. Mean ADI (65.9) and SVI (6.7) differed by 59.2 among the 2.4% (172) of poor index agreement tracts [4b. low SVI (10%), high ADI (40%)].

S1 Fig identifies percentages of tracts in each comparison group out of all 71,724 tracts with available ADI and SVI scores, as well as the number and percentage of urban tracts by agreement comparison group. Tracts in groups 1b. and 4b. accounted for 1.3% (91 tracts) and 2.4% (172 tracts) of Comparison I. and IV. tracts, or 0.1% and 0.2% of all U.S. tracts in the sample (S1 Fig). Group 2b. and 3b. tracts accounted for 19.4% (1391 tracts) and 16.3% (1182 tracts) of Comparison II. and III. tracts, or 1.9% and 1.6% of all U.S. tracts in the sample (S1 Fig). The distribution of good and poor agreement tracts by state is available in S5 and S6 Figs. The highest rates of 2b. and 3b. poor agreement tracts were in California, New York, Massachusetts, New Jersey, Hawaii, Washington, the District of Columbia, and Florida.

### ADI and SVI items driving poor index agreement: Item mean comparisons and mapping

**Fig 4:**
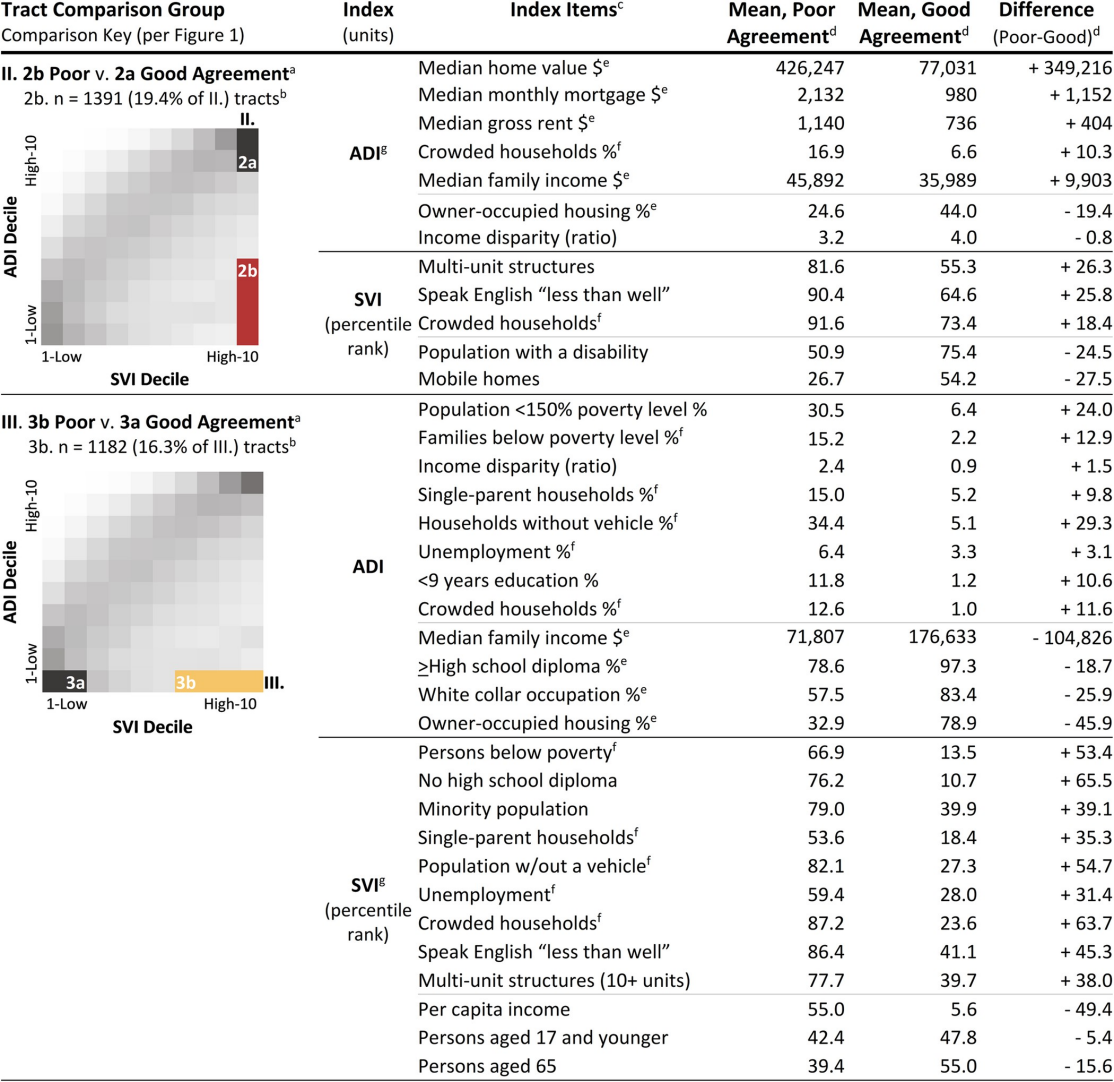
ADI and SVI items driving the 2 highest rates of poor index agreement. *Abbreviations*: ADI, area deprivation index; SVI, social vulnerability index; w/out, without. ^a^ = ADI and SVI items driving poor agreement were identified via item mean comparisons between (see also S5 and S6 Tables): **II) 2b. Poor** [High SVI (10%), Low ADI (40%)] v. **2a. Good** [High SVI (10%), High ADI (20%)] agreement tracts and **III) 3b. Poor** [Low ADI (10%), High SVI (40%)] v. **3a. Good** [Low ADI (10%), Low SVI (20%)] agreement tracts. ^b^ = “n” indicates the number of poor agreement tracts; the percentage denominator is the total comparison decile (II. or III.) tract count. ^c^ = Items with significant (p<0.05) mean comparisons between poor and good agreement tracts and large effect size (Cohen’s D |≥0.80|). ^d^ = Poor index agreement: index score difference of at least 6 deciles; Good index agreement: 0 to 1 decile difference between scores. ADI and (all) SVI items ranging from 0 to 1 were multiplied by 100 for comparisons. ^e^ = Higher item scores indicate higher deprivation levels, except as noted here: lower values indicate higher deprivation. ^f^ = Both ADI and SVI contain this item. ^g^ = Indicates index with items driving poor index agreement.

Fig 4 lists characteristics of tracts–the specific ADI and SVI items–driving the two highest rates of poor index agreement (2b. and 3b. tracts from Fig 3). These poor agreement tracts had low deprivation per ADI and high deprivation according to SVI.

#### Comparison II: ADI and SVI items driving poor agreement

Comparison II tracts had the highest 10% SVI deprivation scores overall, with high poverty, unemployment, minority population, single-parent households, and population without a vehicle, and as well as low per capita income and education (S5 Table). When compared to good agreement tracts [2a. high SVI (10%), high ADI (20%)], the 1,391 (19.4%) poor agreement tracts [2b. high SVI (10%), low ADI (40%)] had significantly (p<0.001; Cohen’s D ≥0.8) higher median home value ($426,247 v. $77,031), median monthly mortgage ($2,132 v. $980), median gross rent ($1,140 v. $736), median family income ($45,892 v. $35,989), multi-unit structures (81.6 v. 55.3 percentile rank), crowded households (16.9% v. 6.6%; 91.6 v. 73.4 percentile rank), and population that speaks English “less than well” (90.4 v. 64.6 percentile rank). Income disparity (3.2 v. 4.0), owner-occupied housing (24.6% v. 44.0%), population with a disability (50.9 v. 75.4 percentile rank), and mobile homes (26.7 v. 54.2 percentile rank) were significantly (p<0.001; Cohen’s D ≥0.8) lower in these tracts. Item comparison results suggested that, in high deprivation tracts according to SVI (10%), poor agreement [low ADI (40%)] was driven by high median home values, median monthly mortgages, and median gross rents.

#### Comparison III: ADI and SVI items driving poor agreement

Comparison III. tracts had the lowest 10% ADI deprivation scores overall, with high median home values, monthly mortgages, and gross rents (S6 Table). When compared to good agreement tracts [3a. low ADI (10%), low SVI (20%)], the 1,182 (16.3%) poor agreement tracts [3b. low ADI (10%), high SVI (40%)] had significantly (p<0.001; Cohen’s D ≥0.8) higher poverty [people below the poverty level (66.9 v. 13.5 percentile rank), <150% of the poverty threshold (30.5% v. 6.4%), and families below the poverty level (15.2% v. 2.2%)], income disparity (2.4 v. 0.9), unemployment (59.4 v. 28.0 percentile rank; 6.5% v. 3.3%), lack of education [no high school diploma (76.2 v. 10.7 percentile rank) and <9 years of education (11.8 v. 1.2%)], minority population (79.0 v. 39.9 percentile rank), single-parent households (53.6 v. 18.4 percentile rank; 15.0% v. 5.2%), households without a vehicle (82.1 v. 27.3 percentile rank; 34.4% v. 5.1%), crowded households (87.2 v. 23.6 percentile rank; 12.6% v. 1.0%), multi-unit structures (77.7 v. 39.7 percentile rank), and people who speak English “less than well” (86.4 v. 41.1 percentile rank). These tracts also had significantly (p<0.001; Cohen’s D ≥0.8) lower median family income ($71,807 v. $176,633), per capita income (55.0 v. 5.6 percentile rank, higher indicated more deprivation), high school graduates (78.6% v. 97.3%), people with white collar occupations (57.5% v. 83.4%), owner-occupied housing 32.9% v. 78.9%), persons 17 years and younger (42.4 v. 47.8 percentile rank), and persons 65 years and older (39.4 v. 55.0 percentile rank). Item comparison results indicated that, in low deprivation tracts according to ADI (10%)–with high median home values, monthly mortgages, and gross rents–poor agreement [high SVI (40%)] was driven by items associated with socioeconomic disadvantage.

#### Mapping

**Fig 5:**
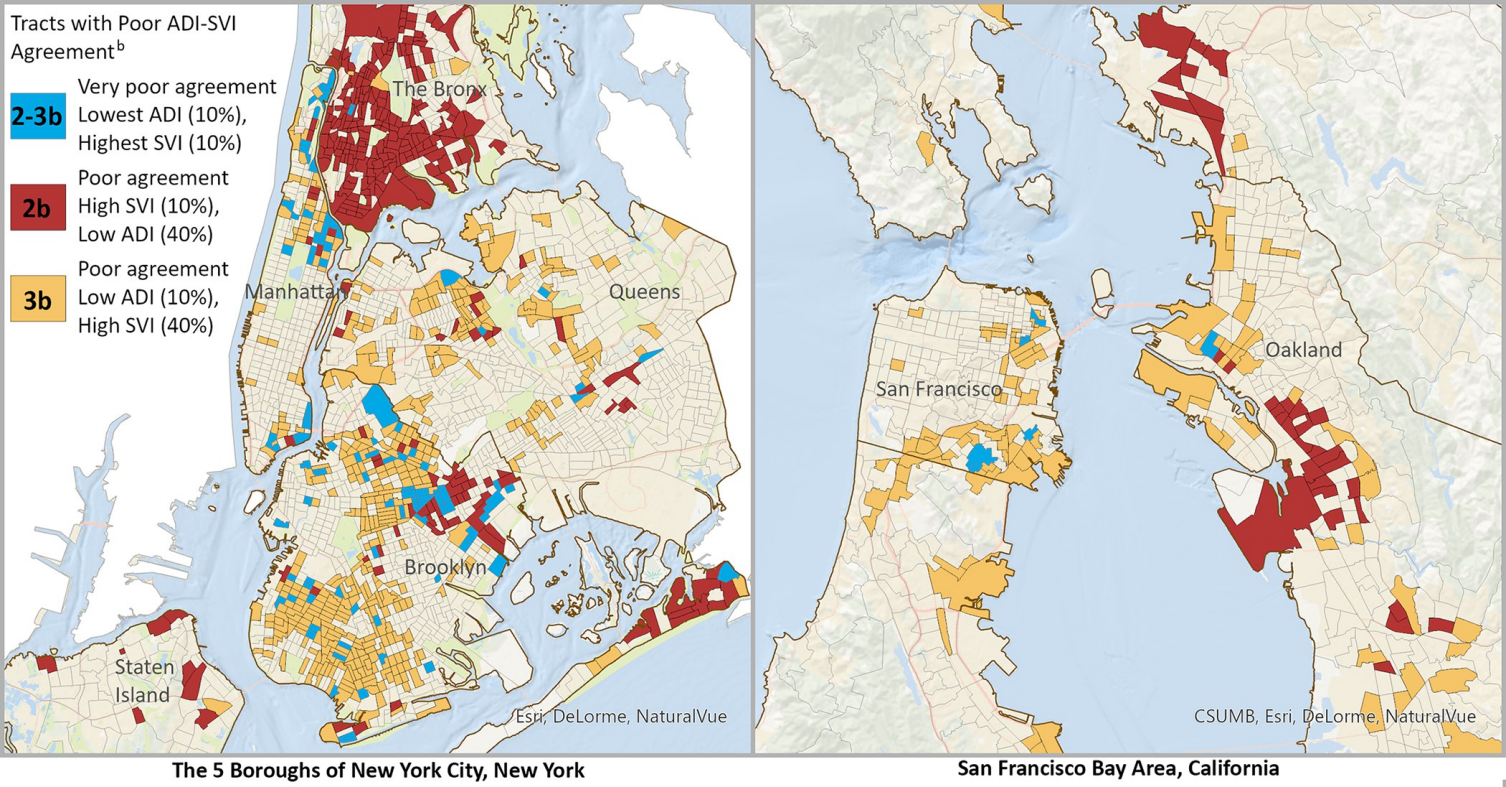
Example locations illustrating high concentrations of tracts with poor index agreement^a^. *Abbreviations*: ADI, area deprivation index; SVI, social vulnerability index. ^a^ = Poor index agreement was defined by ADI and SVI index scores that differed by at least 6 deciles. ^b^ = 2b. and 3b. refer to Fig 3 comparisons of tracts with poor index agreement (with Fig 4 items); “2b-3b” refers to the 164 U.S. tracts that appear in both groups. Data sources: 2010 U.S. Census TIGER/Line shapefiles: state and census tract; 2019 ADI [1]; 2018 SVI [2]; Basemap: Content is the intellectual property of Esri and is used herein with permission. Copyright © 2023 Esri and its licensors. All rights reserved.

In addition to tract characteristics identified via individual ADI and SVI item mean comparisons, one pattern emerged from mapping tracts with the highest rates of poor index agreement (2b. and 3b. tracts, Fig 3). Of the tracts in comparison groups 2b. and 3b., 98.6% and 98.5%, respectively (S1 Fig), were located in urban areas according to Rural Urban Commuting Area codes [25]. Fig 5 maps present 2 example locations with high concentrations of these tracts: the 5 boroughs of New York City and the San Francisco Bay Area. S3 Fig illustrates additional locations containing high numbers of 2b. and 3b. tracts (Miami, Seattle, Los Angeles, San Diego, Sacramento, San Jose, Boston, and Washington, DC). Locations were consistent with urban characteristics identified via item mean comparisons (e.g., more multi-unit structures, more renters, fewer homeowners, less vehicle access, higher minority population, and higher population that speaks English “less than well”).

S4 and S7 Tables contain complete item mean comparison data for poor agreement tracts 1b. and 4b. with high deprivation according to ADI and low deprivation according to SVI. Index items driving poor agreement for these groups are summarized in S2 Fig. Tracts were less likely to be located in urban areas [61.5% (1b.) and 74.4% (4b.)] when compared to groups 2b. and 3b. (S1 Fig). Maps also illustrated that these tracts were primarily located in lower density areas (S4 Fig).

## Discussion

In this comparison of two frequently used area-level socioeconomic deprivation indices, ADI and SVI, 2 principal findings were observed. First, ADI and SVI were overlapping but unique area-level deprivation measures at the tract level. In addition to substantive differences in index purpose, domains, items, construction, scoring method, availability, and geographic scale, index correlation (R = 0.51) and agreement (44.4% of tracts within 0 to 1 deciles) were modest. Nearly 20% of tracts with low ADI and high SVI scores, and 6.3% of all U.S. tracts, had poor index agreement (≥6-decile difference).

Second, tracts with the two highest rates of poor index agreement were more likely to be located in urban areas with higher median monthly mortgages, monthly rents, and home values, while also having characteristics indicative of socioeconomic deprivation (e.g., families in poverty, household crowding, limited education). Such characteristics are often consistent with a process of gentrification and displacement [26–29]. This finding suggested that ADI’s inclusion of unstandardized [30] housing price items can lead to misclassifying these urban tracts as “not deprived” in comparison to SVI, which excluded these items. Taken together, findings highlighted the need for careful selection of a socioeconomic deprivation measure that is sensitive to the context in which it will be used, especially when relying on these measures to identify or focus limited resources on urban areas most in need [6].

Index comparisons from this study expanded upon prior work that documented substantive differences between ADI and SVI and overall modest correlation [3,6,12]. Prior work comparing deprivation indices focused largely on substantive comparisons [1,3,6,7,9,10,31], differences by spatial scale [1,10,12], or larger geographies such as county [8,32,33], clinical referral regions [12], and specific locations or states [32,34]. The present study was the first nation-wide tract level analysis of the ADI and SVI that 1) quantified index agreement at different extremes, providing more detailed insights into the magnitude and specificity of disagreement at specific ADI and SVI levels; 2) identified items driving that agreement; and 3) mapped the distribution of index agreement so that implications of index differences for research, practice, and policy can be examined. Results indicated that the recently documented over-weighting of housing costs in ADI persists in urban areas at the tract level nationally, extending work focused on New York block groups [30,34]. Quantifying, identifying drivers of, and mapping index differences at the tract level across the U.S. is necessary to inform and evaluate research, funding and resource allocation, and policy using these indices. By identifying tracts with poor index agreement, index users can make informed decisions about index selection, plan to address index limitations, and anticipate consequences.

### Implications

Understanding detailed differences between area deprivation indices is necessary for selecting an appropriate index and navigating its limitations. Study findings suggest several considerations for researchers, public health practitioners, healthcare providers and payers, and policy makers using area-level socioeconomic deprivation indices.

#### Index purpose, construction, and application context matter

ADI and SVI were developed for different purposes and capture overlapping but unique constructs: socioeconomic deprivation and social vulnerability. ADI identifies block-group level socioeconomic deprivation to inform program planning, health delivery, and policy [16]. SVI aids emergency response planners and public health officials by identifying areas most likely to require support preparing for, responding to, and recovering from emergencies [18]. Neither index was initially intended for widespread research on associations between public health outcomes and social determinants of health [33]. Implications of index differences, however, can vary by context. Studies have found significant differences in associations between different indices and health outcomes such as pain severity [31] and outcomes related to mortality, physical health, mental health, subjective well-being, and social capital [32]. Other work found no substantial differences in adjusted associations between county-level deprivation according to four area-level socioeconomic deprivation measures (including ADI and SVI) and COVID-19 incidence and mortality rates [8]. While both measures capture disadvantage and vulnerability in meaningful ways, index selection requires an understanding of how and whether index purpose, construction, and usage context (e.g., location, geographic scale, population, and outcomes of interest) affect application.

#### Index differences affect how deprivation is defined and have implications for how indices are used and interpreted, especially within the context of health equity [3]

Deprivation measures used to study relationships between SES and health outcomes can influence predictive ability [31]. Index selection also affects policy and resource allocation decisions, with the potential to divert resources away from areas in need [12,31,34]. For example, using ADI to identify high deprivation areas to inform disaster response, resource allocation, or healthcare payment weighting could result in misclassifying and under-resourcing urban locations with both high deprivation and housing costs [30,34]. Using SVI may raise legal concerns in some contexts due to its inclusion of race and ethnicity [6,8,35]. Strategies to address health equity that rely on these indices must consider index differences, limitations, and application context. Otherwise, strategies may risk maintaining or worsening rather than mitigating inequities.

#### The selected index must include particular aspects of deprivation or social risk relevant to the context in which the index will be used [6]

Indicators of deprivation may vary based on place [10] and a single index may not be appropriate for some contexts. For example, deprivation indicators can vary in rural versus urban settings [36–38]. Results from this study demonstrated that a large proportion of tracts with poor agreement due to ADI housing cost items were located in urban areas with high housing costs and deprivation; a small proportion of tracts with poor agreement were located in less dense and rural areas, with less substantial implications at a national scale. These implications could become more problematic when focusing specifically on these rural areas.

Additionally, prior work found that ADI and SVI items varied in their associations with deprivation, especially race, disability, and household composition items [3]. Historical and contemporary factors, such as structural racism, contribute to place-based differences in resource availability including social capital and material and non-material attributes related to vulnerability [10]. Careful index selection is especially critical for users addressing health equity considering potential links between poor index agreement, urban areas, and documented effects of structural racism [39–41] and gentrification [26,28].

#### Index selection priorities vary for researchers, practitioners, providers and payers, and policy makers

Priorities can range from identifying indices with good construct validity and parsimony, or suited for longitudinal research; to selecting indices to inform disaster response and interventions, locate high deprivation areas, or be used and understood by a variety of audiences; to relying on indices with high potential to inform policy. These priorities require unique considerations and likely result in various index selections for different applications.

No “gold standard” or best index recommendation exists for quantifying socioeconomic disadvantage or social vulnerability. A review of 21 area-level deprivation indices [6], including ADI and SVI, concluded that none are ideal for informing national policies that address social determinants of health or health-related social needs; yet, ADI and SVI were among 3 indices recommended for immediate use in policy development addressing health-related social needs [13]. Once measures and policies are established that determine how resources are distributed, future changes can be difficult to make [6]. Therefore, understanding index differences in detail and identifying the consequences of index selection can enable adjustments so all populations benefit from resource allocation decisions and policies as intended.

### Limitations

This study was not without limitations. The data source years used by the ADI 2019 and SVI 2018 versions differed by one year. Changes in index items over time may have contributed to index agreement results. Indices using census data from 2010–2019 may present additional challenges when used with communities that experienced substantial shifts during the COVID-19 pandemic or natural disasters in 2020 or later [6]. Discrepancies in data source years of available indices must be addressed when selecting, interpreting, and applying area-level deprivation measures. Moreover, changes in deprivation over time, rather than cross-sectional measures of deprivation, may need to be considered [12]. This analysis analyzed the most recent ADI and SVI versions available at the time of the study. Second, this study assessed index agreement using composite scores categorized by deciles and not original continuous rank data. Sensitivity analyses indicated no change in the ADI-SVI correlation coefficient between continuous and decile data.

Third, ADI was developed at the block group rather than tract level. Aggregation of data from block group to tract level may have contributed to index agreement results, although sensitivity analyses in prior work found that aggregation did not change study results [12]. Prior studies also found that differences between indices and associated health outcomes vary across geographic scales, with larger differences occurring at smaller geographic areas (e.g., census block groups) and decreasing differences at larger geographic areas (e.g., county) [5,8,32,33]. This work suggests that index differences may be underestimated at tract compared to the block group level. Finally, study findings were based on comparisons of ADI and SVI data across all U.S. census tracts. Repeating the comparisons within specific states, using state-level ADI and SVI data, may reveal additional information about index agreement within states. The present study’s nationwide analysis is timely, however, given the widespread use of ADI and SVI with U.S. datasets and recommended use for immediate national policy development [6,13].

## Conclusions

ADI and SVI are not interchangeable measures. They each include unique domains of socioeconomic deprivation that affect index agreement, especially in urban areas with both high housing costs and characteristics indicative of socioeconomic deprivation. Study findings emphasized the need for intentional selection of an area-level deprivation index by researchers, practitioners, and policymakers working to address health equity. The measure must be appropriately sensitive to the context in which it will be used.

## Supporting information

S1 Fig Characteristics of tract comparison groups by good and poor index agreement. (PDF)

S2 Fig Characteristics of tracts with the 2 lowest rates of poor index agreement. (PDF)

S3 Fig Example locations of tracts with poor ADI-SVI agreement, comparisons 2b & 3b. (PDF)

S4 Fig Example locations of tracts with poor ADI-SVI agreement, comparisons 1b & 4b. (PDF)

S5 Fig Distribution of tracts with good agreement by state. (PDF)

S6 Fig Distribution of tracts with poor agreement by state. (PDF)

S1 Table Summary of ADI and SVI characteristics. (PDF)

S2 Table ADI and SVI domain and item summary. (PDF)

S3 Table ADI and SVI item descriptive statistics. (PDF)

S4 Table Individual index item mean comparisons by Agreement: I. High ADI (10%). (PDF)

S5 Table Individual index item mean comparisons by Agreement: II. High SVI (10%). (PDF)

S6 Table Individual index item mean comparisons by Agreement: III. Low ADI (10%). (PDF)

S7 Table Individual index item mean comparisons by Agreement: IV. Low SVI (10%). (PDF)

S1 File Supporting information references. (PDF)
