# Supplementary material for: Comparison of two area-level socioeconomic deprivation indices: Implications for public health research, practice, and policy
Source: PLoS One. 2023 Oct 5;18(10):e0292281. doi: 10.1371/journal.pone.0292281 (PMC10553799; doi:10.1371/journal.pone.0292281)
Supplement: S1 Fig — (PDF) [file pone.0292281.s001.pdf]

Figure S1. Characteristics of Tract Comparison Groups by Good and Poor Index Agreement

Tract Comparison Group Key[a] (per Figure 3)

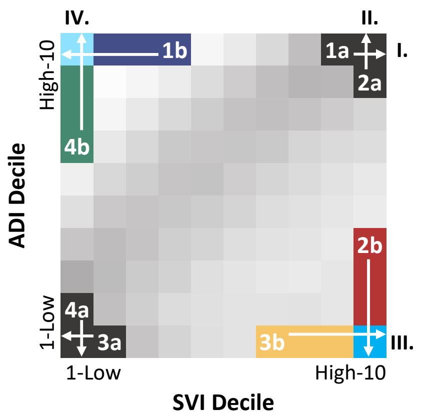

	N[b] (tracts in deciles I.-IV.)	**Tract Comparison Group, Index Agreement,**[c] Agreement Definition	**n,** tracts # (% of N)	**% of 71,724 tracts**[d]	**Urban tracts** # (% of n)[d]
**I.**	6771	**1a. Good Agreement,** High ADI (10%), High SVI (20%)	4294 (63.4)	6.0	3302 (76.9)
		**1b. Poor Agreement,** High ADI (10%), Low SVI (40%)	91 (1.3)	0.1	56 (61.5)
**II.**	7188	**2a. Good Agreement,** High SVI (10%), High ADI (20%)	3655 (50.9)	5.1	2854 (78.1)
		**2b. Poor Agreement,** High SVI (10%), Low ADI (40%)	1391 (19.4)	1.9	1372 (98.6)
**III.**	7262	**3a. Good Agreement,** Low ADI (10%), Low SVI (20%)	3183 (43.8)	4.4	3128 (98.3)
		**3b. Poor Agreement,** Low ADI (10%), High SVI (40%)	1182 (16.3)	1.6	1164 (98.5)
**IV.**	7160	**4a. Good Agreement,** Low SVI (10%), Low ADI (20%)	3605 (50.3)	5.0	3546 (98.4)
		**4b. Poor Agreement,** Low SVI (10%), High ADI (40%)	172 (2.4)	0.2	128 (74.4)

Abbreviations: ADI, area deprivation index; SVI, social vulnerability index.
[a] = The image illustrates the 8 census tract comparison groups with respect to the distribution of tracts by ADI and SVI decile scores shown in Figure 1.
[b] = N indicates the total number of tracts within a comparison decile, the first decile noted in the agreement definition [e.g., I. 6,771 total tracts in the top ADI (10%) decile].
[c] = Good index agreement was defined as index scores that differed by 0 to 1 decile. Poor index agreement was defined as index scores that differed by at least 6 deciles.
[d] = Percentage of the comparison group tracts ("n") out of the 71,724 U.S. census tracts with both ADI and SVI scores (74,001 total).
[e] = Number and percentage of "n" tracts that were classified as "urban" and not rural according to the 2010 Rural Urban Commuting Area codes.
