# Supplementary material for: Comparison of two area-level socioeconomic deprivation indices: Implications for public health research, practice, and policy
Source: PLoS One. 2023 Oct 5;18(10):e0292281. doi: 10.1371/journal.pone.0292281 (PMC10553799; doi:10.1371/journal.pone.0292281)
Supplement: S2 Fig — (PDF) [file pone.0292281.s002.pdf]

Figure S2. Characteristics of Tracts with the 2 Lowest Rates of Poor Index Agreement

I. 1b Poor v. **1a Good Agreement**[a]
1b. n = 91 (1.3% of I.) tracts[b]

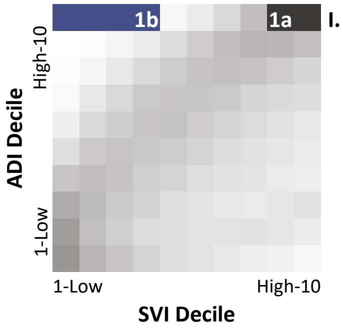

Tract Comparison Group<br>Comparison Key (per Figure S1)	Index<br>(units)	Index Item[c]	Mean, Poor Agreement[d]	Mean, Good Agreement[d]	Difference (Poor-Good)[d]
I. 1b Poor v. 1a Good Agreement[a]	ADI	≥High school diploma %[e]	90.0	74.3	+ 15.78
	ADI	Median family income $[e]	53,383	35,632	+ 17,751
	ADI	White collar occupation %[e]	52.5	39.4	+ 13.1
	ADI	Owner-occupied housing %[e]	69.5	46.5	+ 23.0
	ADI	Population <150% poverty level %	30.3	51.3	- 21.0
	ADI	<9 years of education %	3.0	10.2	- 7.2
	ADI	Crowded households %[f]	0.7	5.2	- 4.5
	ADI	Families below poverty level %[f]	12.1	30.3	- 18.3
	ADI	Income disparity (ratio)	2.9	4.0	- 1.2
	ADI	Households w/out vehicle %[f]	6.1	19.5	- 13.4
	ADI	Single-parent households %[f]	12.9	29.9	- 17.0
	ADI	Unemployment %[f]	5.1	11.9	- 6.8
	ADI	Median gross rent $[e]	711	721	- 9
	ADI	Households w/out a telephone %	2.0	3.6	- 1.5
	SVI[g] (percentile rank)	Per capita income	61.6	90.6	+ 29.0
	SVI[g] (percentile rank)	No high school diploma	44.0	84.5	- 40.6
	SVI[g] (percentile rank)	Persons below poverty[f]	59.8	90.4	- 30.6
	SVI[g] (percentile rank)	Crowded households[f]	15.2	65.3	- 50.1
	SVI[g] (percentile rank)	Population w/out a vehicle[f]	45.6	81.2	- 35.5
	SVI[g] (percentile rank)	Speak English "less than well"	15.3	53.2	- 37.9
	SVI[g] (percentile rank)	Minority population	22.7	78.7	- 55.9
	SVI[g] (percentile rank)	Single-parent households[f]	40.1	82.9	- 42.8
	SVI[g] (percentile rank)	Unemployment[f]	40.4	82.2	- 41.8
	SVI[g] (percentile rank)	Multi-unit structures (10- units)	24.1	49.0	- 24.9
	SVI[g] (percentile rank)	Population with a disability	68.4	78.4	- 10.0
IV. 4b Poor v. 4a Good Agreement[a]	ADI[g]	Population <150% poverty level %	13.7	5.7	+ 8.0
	ADI[g]	Families below poverty level %[f]	4.7	2.0	+ 2.7
	ADI[g]	Single-parent households %[f]	8.6	5.0	+ 3.5
	ADI[g]	Income disparity (ratio)	1.6	0.8	+ 0.8
	ADI[g]	Median monthly mortgage $[e]	1,163	2,889	- 1,726
	ADI[g]	White collar occupation %[e]	61.0	81.7	- 20.7
	ADI[g]	Median family income $[e]	76,486	162,975	- 86,490
	ADI[g]	Median gross rent $[e]	874	1,947	- 1,073
	ADI[g]	≥High school diploma %[e]	94.6	97.5	- 2.9
	ADI[g]	Median home value $[e]	127,678	632,912	- 505,235
	SVI (percentile rank)	Per capita income	36.1	7.3	+ 28.9
	SVI (percentile rank)	Population with a disability	43.9	15.4	+ 28.5
	SVI (percentile rank)	No high school diploma	25.2	9.5	+ 15.7
	SVI (percentile rank)	Persons below poverty[f]	25.8	10.9	+ 14.9
	SVI (percentile rank)	Minority population	16.4	32.4	- 16.0
	SVI (percentile rank)	Speak English "less than well"	13.9	32.4	- 18.5

IV. 4b Poor v. **4a Good Agreement**[a]
4b. n = 172 (2.4% of IV.) tracts[b]

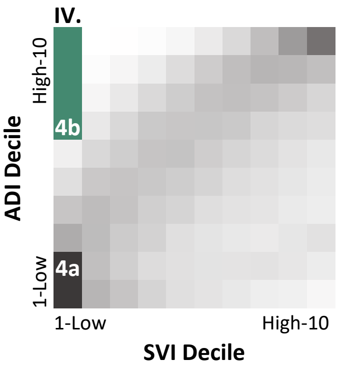

Abbreviations: ADI, area deprivation index; SVI, social vulnerability index; w/out, without.

[a] = Poor agreement tract characteristics were identified via item mean comparisons between (see also SI Tables S4 and S7):
I) 1b. Poor [High ADI (10%), Low SVI (40%)] v. **1a. Good** [High ADI (10%), High SVI (20%)] agreement tracts and
IV) 4b. Poor [Low SVI (10%), High ADI (40%)] v. **4a. Good** [Low SVI (10%), Low ADI (20%)] agreement tracts.

[b] = "n" indicates the number of poor agreement tracts; the percentage denominator is the total comparison decile (I. or IV.) tract count.

[c] = Items with significant ($p<0.05$) mean comparisons between poor and good agreement tracts and large effect size (Cohen's D $|\geq 0.80|$).

[d] = Poor index agreement: index score difference of at least 6 deciles; Good index agreement: 0 to 1 decile difference between scores. ADI and (all) SVI items ranging from 0 to 1 were multiplied by 100 for comparisons.

[e] = Higher item scores indicate higher deprivation levels, except as noted here: lower values indicate higher deprivation.

[f] = Both ADI and SVI contain this item.

[g] = Indicates index item characteristics driving poor index agreement.
