# Supplementary material for: Comparison of two area-level socioeconomic deprivation indices: Implications for public health research, practice, and policy
Source: PLoS One. 2023 Oct 5;18(10):e0292281. doi: 10.1371/journal.pone.0292281 (PMC10553799; doi:10.1371/journal.pone.0292281)
Supplement: S3 Fig — (PDF) [file pone.0292281.s003.pdf]

Rollings, K.A., Noppert, G.A., Griggs, J.J., Melendez, R.A., Clarke, P.J. (2023). Comparison of two area-level socioeconomic deprivation indices: Implications for public health research, practice, and policy. *PLoS One*. 10.1371/journal.pone.0292281

Figure S3. Example Locations of Tracts with Poor ADI-SVI Agreement, Comparisons 2b & 3b[a]

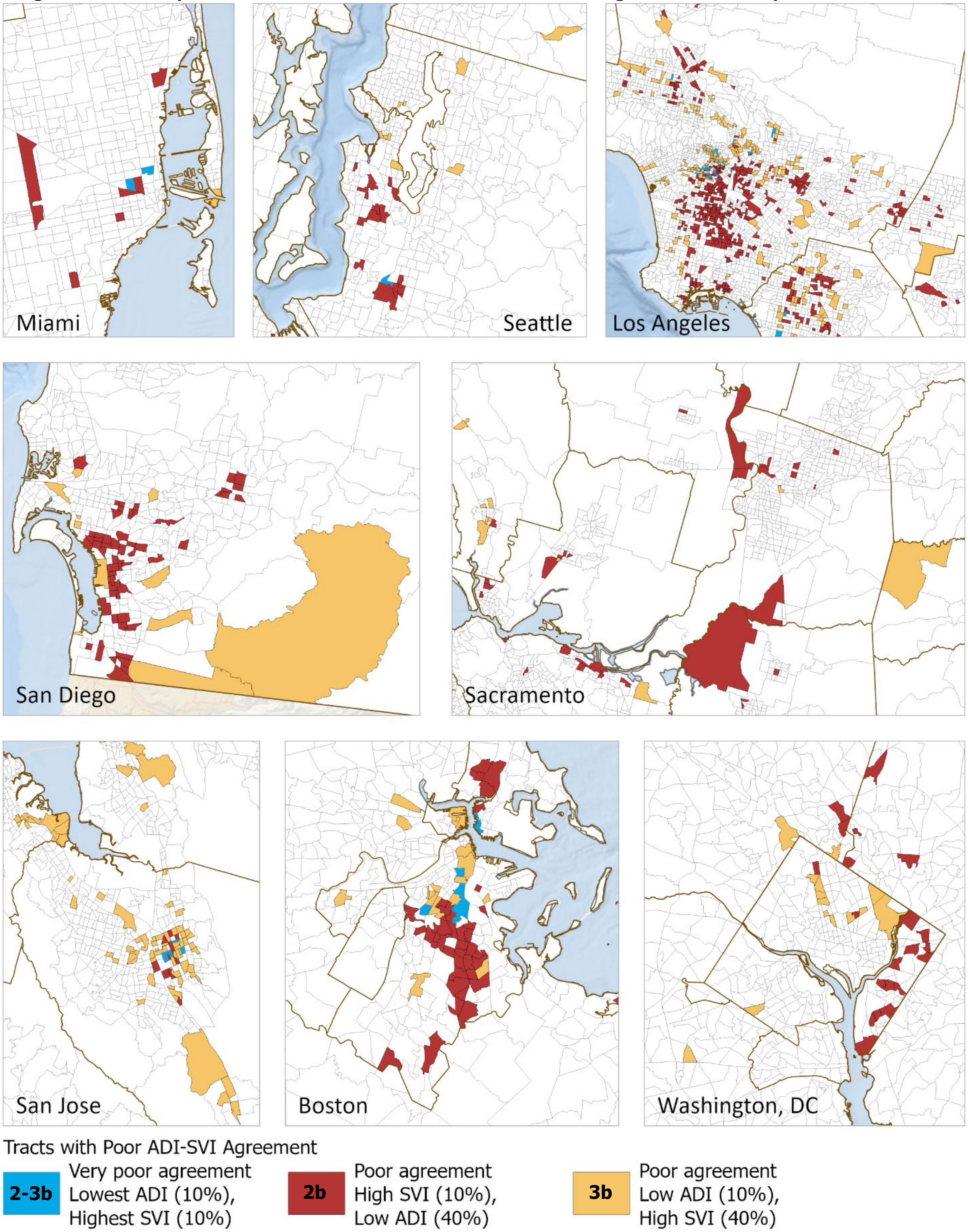

Abbreviations: ADI, area deprivation index; SVI, social vulnerability index.

[a] = Poor index agreement was defined by ADI and SVI index scores that differed by at least 6 deciles.

[b] = 2b. and 3b. refer to Figure 3 comparisons of tracts with poor index agreement.

Data sources: 2010 U.S. Census TIGER/Line shapefiles: state and census tract; 2019 ADI [1]; 2018 SVI [2]; *Basemap*: Content is the intellectual property of Esri and is used herein with permission. Copyright © 2023 Esri and its licensors. All rights reserved.
