# Supplementary material for: Comparison of two area-level socioeconomic deprivation indices: Implications for public health research, practice, and policy
Source: PLoS One. 2023 Oct 5;18(10):e0292281. doi: 10.1371/journal.pone.0292281 (PMC10553799; doi:10.1371/journal.pone.0292281)
Supplement: S4 Fig — (PDF) [file pone.0292281.s004.pdf]

Figure S4. Example Locations of Tracts with Poor ADI-SVI Agreement, Comparisons 1b & 4b[a]

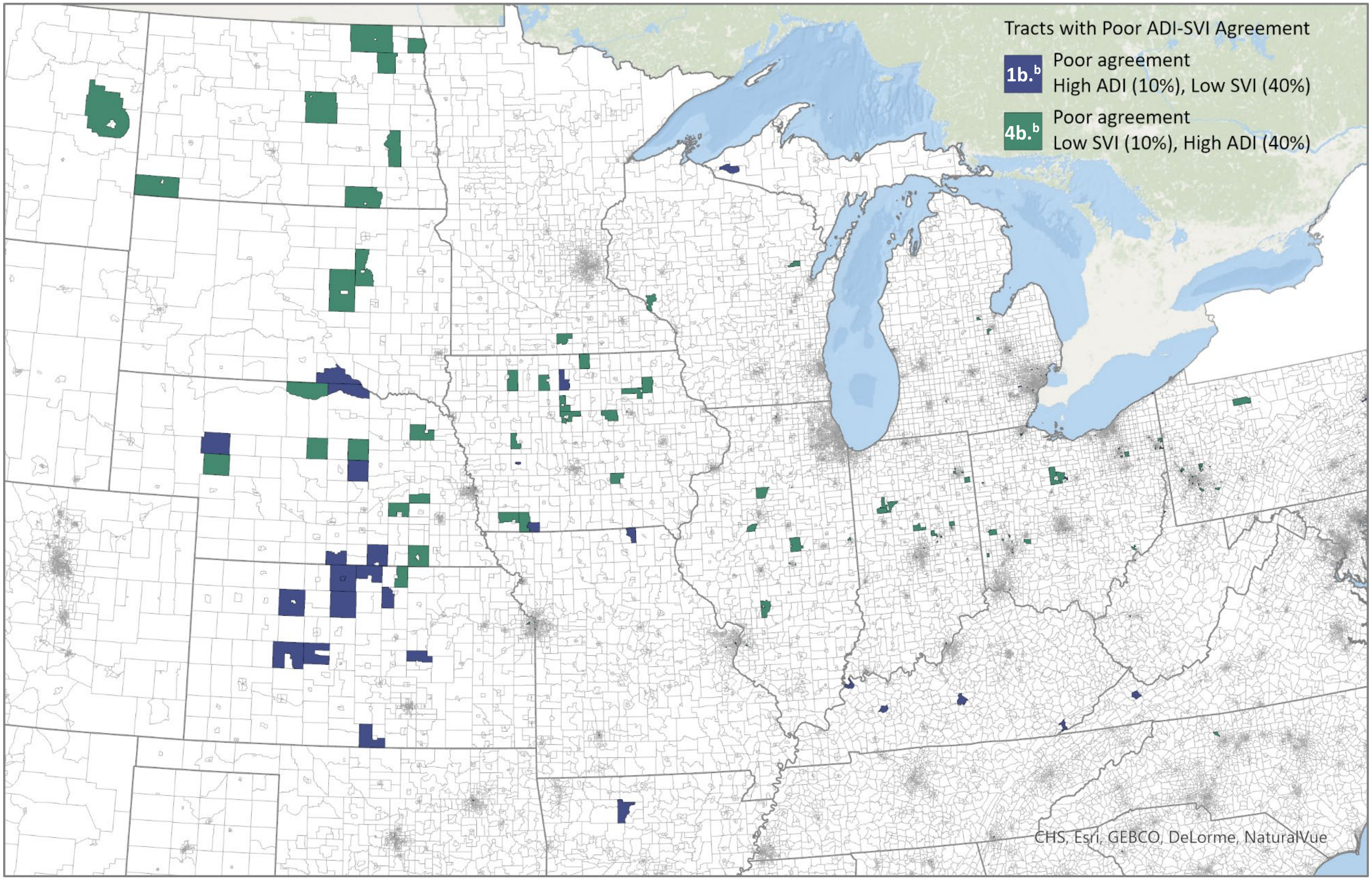

Abbreviations: ADI, area deprivation index; SVI, social vulnerability index.
[a] = Poor index agreement was defined by ADI and SVI index scores that differed by at least 6 deciles.
[b] = 1b. and 4b. refer to Figure 3 comparisons of tracts with poor index agreement.
Data sources: 2010 U.S. Census TIGER/Line shapefiles: state and census tract; 2019 ADI [1]; 2018 SVI [2];
Basemap: Content is the intellectual property of Esri and is used herein with permission. Copyright © 2023 Esri and its licensors. All rights reserved.
