# Supplementary material for: Comparison of two area-level socioeconomic deprivation indices: Implications for public health research, practice, and policy
Source: PLoS One. 2023 Oct 5;18(10):e0292281. doi: 10.1371/journal.pone.0292281 (PMC10553799; doi:10.1371/journal.pone.0292281)
Supplement: S5 Fig — (PDF) [file pone.0292281.s005.pdf]

Figure S5. Distribution of Tracts with Good Agreement by State

	**Good Agreement Tract Comparison Group** (per Figure 3)				**Total, good**	**Rank, good agreement tracts**				
**State**	**1a** (% n)	**2a** (% n)	**3a** (% n)	**4a** (% n)	**agreement tracts**	**Total**	**1a**	**2a**	**3a**	**4a**
	n=4,294 tracts	n=3,655 tracts	n=3,183 tracts	n=3,605 tracts	n=14,737 tracts	(1=highest count)				
Texas	680(15.8)	688(18.8)	144(4.5)	245(6.8)	1757	1	1	1	6	2
California	31(0.7)	92(2.5)	960(30.2)	449(12.5)	1532	2	25	15	1	1
New York	160(3.7)	128(3.5)	296(9.3)	213(5.9)	797	3	10	9	2	5
Florida	187(4.4)	203(5.6)	112(3.5)	157(4.4)	659	4	5	3	10	10
Michigan	350(8.2)	194(5.3)	27(0.8)	77(2.1)	648	5	2	4	21	17
Illinois	160(3.7)	127(3.5)	126(4.0)	181(5.0)	594	6	10	10	8	8
Pennsylvania	205(4.8)	171(4.7)	72(2.3)	136(3.8)	584	7	4	6	12	11
Ohio	317(7.4)	176(4.8)	12(0.4)	53(1.5)	558	8	3	5	27	21
Virginia	35(0.8)	37(1.0)	202(6.3)	226(6.3)	500	9	24	26	4	3
North Carolina	162(3.8)	204(5.6)	38(1.2)	85(2.4)	489	10	8	2	17	16
Georgia	187(4.4)	157(4.3)	40(1.3)	91(2.5)	475	11	5	8	15	14
New Jersey	26(0.6)	34(0.9)	208(6.5)	201(5.6)	469	12	28	27	3	6
Arizona	115(2.7)	163(4.5)	40(1.3)	93(2.6)	411	13	16	7	15	13
Massachusetts	2(0.0)	5(0.1)	183(5.7)	175(4.9)	365	14	42	40	5	9
Colorado	16(0.4)	20(0.5)	113(3.6)	215(6.0)	364	15	32	30	9	4
Maryland	27(0.6)	24(0.7)	109(3.4)	185(5.1)	345	16	26	29	11	7
Tennessee	126(2.9)	79(2.2)	28(0.9)	59(1.6)	292	17	14	18	20	20
Indiana	174(4.1)	91(2.5)	4(0.1)	14(0.4)	283	18	7	17	35	33
Alabama	161(3.7)	97(2.7)	6(0.2)	13(0.4)	277	19	9	14	32	35
Louisiana	114(2.7)	121(3.3)	11(0.3)	18(0.5)	264	20	17	11	28	29
South Carolina	114(2.7)	98(2.7)	19(0.6)	33(0.9)	264	20	17	13	24	24
Washington	3(0.1)	18(0.5)	128(4.0)	111(3.1)	260	22	39	32	7	12
Oklahoma	140(3.3)	92(2.5)	4(0.1)	14(0.4)	250	23	12	15	35	33
Mississippi	134(3.1)	105(2.9)	0(0.0)	1(0.0)	240	24	13	12	44	49
Missouri	118(2.7)	58(1.6)	13(0.4)	31(0.9)	220	25	15	22	26	25
Wisconsin	82(1.9)	64(1.8)	7(0.2)	30(0.8)	183	26	21	21	30	26
Kentucky	97(2.3)	52(1.4)	6(0.2)	18(0.5)	173	27	20	23	32	29
Arkansas	101(2.4)	66(1.8)	2(0.1)	3(0.1)	172	28	19	20	39	44
Connecticut	4(0.1)	16(0.4)	60(1.9)	89(2.5)	169	29	37	33	13	15
Kansas	72(1.7)	45(1.2)	7(0.2)	26(0.7)	150	30	22	24	30	27
New Mexico	53(1.2)	72(2.0)	4(0.1)	12(0.3)	141	31	23	19	35	36
Nevada	23(0.5)	41(1.1)	18(0.6)	35(1.0)	117	32	29	25	25	23
Minnesota	7(0.2)	12(0.3)	20(0.6)	77(2.1)	116	33	34	35	23	17
Utah	2(0.0)	3(0.1)	27(0.8)	72(2.0)	104	34	42	43	21	19
Oregon	2(0.0)	4(0.1)	37(1.2)	40(1.1)	83	35	42	42	18	22
Nebraska	19(0.4)	26(0.7)	2(0.1)	12(0.3)	59	36	31	28	39	36
Hawaii	0(0.0)	0(0.0)	45(1.4)	11(0.3)	56	37	48	47	14	39
Iowa	27(0.6)	19(0.5)	0(0.0)	3(0.1)	49	38	26	31	44	44
District of	0(0.0)	0(0.0)	31(1.0)	15(0.4)	46	39	48	47	19	31
New Hampshire	0(0.0)	0(0.0)	4(0.1)	26(0.7)	30	40	48	47	35	27
South Dakota	13(0.3)	14(0.4)	0(0.0)	3(0.1)	30	40	33	34	44	44
West Virginia	21(0.5)	5(0.1)	0(0.0)	1(0.0)	27	42	30	40	44	49
Delaware	2(0.0)	2(0.1)	8(0.3)	12(0.3)	24	43	42	46	29	36
Rhode Island	1(0.0)	3(0.1)	5(0.2)	15(0.4)	24	43	46	43	34	31
Montana	6(0.1)	7(0.2)	2(0.1)	7(0.2)	22	45	36	36	39	40
Alaska	4(0.1)	7(0.2)	2(0.1)	5(0.1)	18	46	37	36	39	43
Idaho	3(0.1)	6(0.2)	0(0.0)	6(0.2)	15	47	39	38	44	42
North Dakota	7(0.2)	6(0.2)	0(0.0)	2(0.1)	15	47	34	38	44	47
Maine	3(0.1)	3(0.1)	0(0.0)	7(0.2)	13	49	39	43	44	40
Vermont	0(0.0)	0(0.0)	1(0.0)	2(0.1)	3	50	48	47	43	47
Wyoming	1(0.0)	0(0.0)	0(0.0)	0(0.0)	1	51	46	47	44	51
