# Supplementary material for: Comparison of two area-level socioeconomic deprivation indices: Implications for public health research, practice, and policy
Source: PLoS One. 2023 Oct 5;18(10):e0292281. doi: 10.1371/journal.pone.0292281 (PMC10553799; doi:10.1371/journal.pone.0292281)
Supplement: S6 Fig — (PDF) [file pone.0292281.s006.pdf]

Figure S6. Distribution of Tracts with Poor Agreement by State

State	Poor Agreement Tract Comparison Group (per Figure 3)				Total, poor agreement tracts	Rank, poor agreement tracts				
	1b (% n) n=91 tracts	2b (% n) n=1,391 tracts	3b (% n) n=1,182 tracts	4b (% n) n=172 tracts	n=2,836 tracts	Total	1b	2b	3b	4b
						(1=highest count)				
California	0 (0.0)	725 (52.1)	511 (43.2)	0 (0.0)	1236	1	21	1	2	30
New York	0 (0.0)	401 (28.8)	550 (46.5)	10 (5.8)	961	2	21	2	1	6
Massachusetts	0 (0.0)	57 (4.1)	21 (1.8)	0 (0.0)	78	3	21	3	4	30
Ohio	17 (18.7)	3 (0.2)	0 (0.0)	29 (16.9)	49	4	1	21	26	1
New Jersey	0 (0.0)	44 (3.2)	3 (0.3)	1 (0.6)	48	5	21	4	10	20
Hawaii	0 (0.0)	9 (0.6)	37 (3.1)	0 (0.0)	46	6	21	10	3	30
Florida	8 (8.8)	19 (1.4)	5 (0.4)	6 (3.5)	38	7	4	6	7	11
Pennsylvania	10 (11.0)	4 (0.3)	2 (0.2)	22 (12.8)	38	7	3	17	13	2
Washington	0 (0.0)	21 (1.5)	12 (1.0)	0 (0.0)	33	9	21	5	5	30
District of Columbia	0 (0.0)	15 (1.1)	10 (0.8)	0 (0.0)	25	10	21	8	6	30
Indiana	8 (8.8)	0 (0.0)	0 (0.0)	16 (9.3)	24	11	4	35	26	3
Texas	1 (1.1)	16 (1.2)	5 (0.4)	2 (1.2)	24	11	16	7	7	15
Michigan	5 (5.5)	2 (0.1)	0 (0.0)	13 (7.6)	20	13	6	24	26	4
Illinois	2 (2.2)	5 (0.4)	3 (0.3)	9 (5.2)	19	14	11	13	10	8
Iowa	4 (4.4)	0 (0.0)	0 (0.0)	13 (7.6)	17	15	8	35	26	4
Kansas	12 (13.2)	0 (0.0)	0 (0.0)	3 (1.7)	15	16	2	35	26	13
Nebraska	5 (5.5)	0 (0.0)	0 (0.0)	10 (5.8)	15	16	6	35	26	6
Oregon	0 (0.0)	10 (0.7)	1 (0.1)	0 (0.0)	11	18	21	9	18	30
Arizona	3 (3.3)	4 (0.3)	1 (0.1)	2 (1.2)	10	19	10	17	18	15
Maryland	0 (0.0)	7 (0.5)	2 (0.2)	1 (0.6)	10	19	21	11	13	20
Missouri	2 (2.2)	0 (0.0)	1 (0.1)	7 (4.1)	10	19	11	35	18	9
Tennessee	1 (1.1)	6 (0.4)	0 (0.0)	1 (0.6)	8	22	16	12	26	20
Georgia	0 (0.0)	5 (0.4)	2 (0.2)	0 (0.0)	7	23	21	13	13	30
Nevada	0 (0.0)	4 (0.3)	2 (0.2)	1 (0.6)	7	23	21	17	13	20
North Carolina	0 (0.0)	4 (0.3)	1 (0.1)	2 (1.2)	7	23	21	17	18	15
North Dakota	0 (0.0)	0 (0.0)	0 (0.0)	7 (4.1)	7	23	21	35	26	9
Virginia	1 (1.1)	2 (0.1)	4 (0.3)	0 (0.0)	7	23	16	24	9	30
Colorado	0 (0.0)	5 (0.4)	1 (0.1)	0 (0.0)	6	28	21	13	18	30
Kentucky	4 (4.4)	1 (0.1)	1 (0.1)	0 (0.0)	6	28	8	29	18	30
Louisiana	2 (2.2)	3 (0.2)	0 (0.0)	1 (0.6)	6	28	11	21	26	20
South Carolina	0 (0.0)	2 (0.1)	3 (0.3)	1 (0.6)	6	28	21	24	10	20
Alabama	2 (2.2)	0 (0.0)	2 (0.2)	1 (0.6)	5	32	11	35	13	20
Connecticut	0 (0.0)	5 (0.4)	0 (0.0)	0 (0.0)	5	32	21	13	26	30
Minnesota	0 (0.0)	3 (0.2)	1 (0.1)	1 (0.6)	5	32	21	21	18	20
Arkansas	1 (1.1)	1 (0.1)	1 (0.1)	1 (0.6)	4	35	16	29	18	20
Oklahoma	0 (0.0)	0 (0.0)	0 (0.0)	4 (2.3)	4	35	21	35	26	12
West Virginia	2 (2.2)	0 (0.0)	0 (0.0)	2 (1.2)	4	35	11	35	26	15
South Dakota	1 (1.1)	0 (0.0)	0 (0.0)	2 (1.2)	3	38	16	35	26	15
Wisconsin	0 (0.0)	0 (0.0)	0 (0.0)	3 (1.7)	3	38	21	35	26	13
Alaska	0 (0.0)	2 (0.1)	0 (0.0)	0 (0.0)	2	40	21	24	26	30
New Mexico	0 (0.0)	2 (0.1)	0 (0.0)	0 (0.0)	2	40	21	24	26	30
Delaware	0 (0.0)	1 (0.1)	0 (0.0)	0 (0.0)	1	42	21	29	26	30
Maine	0 (0.0)	1 (0.1)	0 (0.0)	0 (0.0)	1	42	21	29	26	30
Mississippi	0 (0.0)	1 (0.1)	0 (0.0)	0 (0.0)	1	42	21	29	26	30
Montana	0 (0.0)	0 (0.0)	0 (0.0)	1 (0.6)	1	42	21	35	26	20
Rhode Island	0 (0.0)	1 (0.1)	0 (0.0)	0 (0.0)	1	42	21	29	26	30
Idaho	0 (0.0)	0 (0.0)	0 (0.0)	0 (0.0)	0	47	21	35	26	30
New Hampshire	0 (0.0)	0 (0.0)	0 (0.0)	0 (0.0)	0	47	21	35	26	30
Utah	0 (0.0)	0 (0.0)	0 (0.0)	0 (0.0)	0	47	21	35	26	30
Vermont	0 (0.0)	0 (0.0)	0 (0.0)	0 (0.0)	0	47	21	35	26	30
Wyoming	0 (0.0)	0 (0.0)	0 (0.0)	0 (0.0)	0	47	21	35	26	30
