# Supplementary material for: Comparison of two area-level socioeconomic deprivation indices: Implications for public health research, practice, and policy
Source: PLoS One. 2023 Oct 5;18(10):e0292281. doi: 10.1371/journal.pone.0292281 (PMC10553799; doi:10.1371/journal.pone.0292281)
Supplement: S1 Table — (PDF) [file pone.0292281.s007.pdf]

Table S1. Summary of ADI and SVI Characteristics

Characteristic	Area Deprivation Index (ADI) [1]	Social Vulnerability Index (SVI) [2]
**Purpose**	To provide relative rankings of neighborhood disadvantage to inform research, program planning, health delivery, and policy especially, for disadvantaged neighborhoods [3, 4].	To spatially identify vulnerable communities and understand the risk of hazards to these populations before, during, and after natural disasters and hazardous events [2, 3, 5, 6].
**Geographic scale**	Census block group[b]	Census tract[a]<br>County
**Index-defined domains**	1. Income<br>2. Education<br>3. Employment<br>4. Housing quality	1. SES<br>2. Household composition and disability<br>3. Minority status and language<br>4. Housing type and transportation
**Index construction**	Block groups are ranked 1-100 based on national percentile rankings, and in deciles from 1-10 for each state. A rank of 100 represents the highest disadvantage level and a rank of 1 indicates the lowest level.	Geographic areas are ranked 0-1 based on percentile rankings. Higher values indicate increased vulnerability.
**Weighting of items and domains**	Weighting is based on factor score coefficients for individual items. All items/domains are weighted and summed. Sums are then transformed into rankings by arbitrarily setting the index mean at 100 and SD at 20.	Items are equally weighted (acceptable to omit variables).
**No. of items**	17	15
**Ranking geography**	National<br>State	National<br>State
**Data source**	American Community Survey 5-Year Estimates, US Census Bureau	American Community Survey 5-Year Estimates, US Census Bureau
**Available years**	2015, 2020, 2021<br>(other years available upon request)	2000, 2010, 2014, 2016, 2018, 2020
**Data update frequency**	Varies	Varies
**Limitations**	Limitations of the American Community Survey persist (e.g., limited accounting for undocumented-immigrant populations) [1, 6].	Census data limitations
**link**	https://www.neighborhoodatlas.medicine.wisc.edu	https://www.atsdr.cdc.gov/placeandhealth/svi/index.html

Note. SES, socioeconomic status

[a] = A census tract contains 1200-8000 people, with an average population of 4,000 per tract.

[b] = A census block group contains 600 to 3000 people.
