# Supplementary material for: Comparison of two area-level socioeconomic deprivation indices: Implications for public health research, practice, and policy
Source: PLoS One. 2023 Oct 5;18(10):e0292281. doi: 10.1371/journal.pone.0292281 (PMC10553799; doi:10.1371/journal.pone.0292281)
Supplement: S2 Table — (PDF) [file pone.0292281.s008.pdf]

Table S2. ADI and SVI Domain and Item Summary

Domain	Index Item	Description
**ADI 2019**[a]		*% Percentage (except as noted)*
**Income**	Families below poverty level[b]	Percentage of families below the poverty level
	Population <150% poverty level	Percentage of population below 150% of the poverty threshold
	Median family income $[c]	Median family income [in U.S. dollars]
	Income disparity (ratio)	Log of 100 x the ratio of the number of households with <$10,000 in income to the number of households with ≥$50,000 in income[4, 5]
**Employment**	Unemployment[b]	Percentage of civilian labor force population aged ≥16 years unemployed
	White collar occupation[c]	Percentage of employed persons aged ≥16 years in white collar occupations
**Education**	<9 years of education	Percentage of population aged ≥25 years with <9 years of education
	≥High school diploma[c]	Percentage of population aged ≥25 years with ≥high school diploma
**Housing**	Owner-occupied housing[c]	Percentage of owner-occupied housing units
	Median monthly mortgage $[c]	Median monthly mortgage [in U.S. dollars]
	Median gross rent $[c]	Median gross rent [in U.S. dollars]
	Median home value $[c]	Median home value [in U.S. dollars]
	Single-parent households[b]	Percentage of occupied housing units w/out complete plumbing
**Household Characteristics**	Households w/out vehicle[b]	Percentage of single-parent households with children <18 years
	Households w/out a telephone	Percentage of households without a motor vehicle
	Households w/ incomplete plumbing	Percentage of households without a telephone
	Crowded households[b]	Percentage of households with >1 person per room
**SVI 2018**[d]		*Percentile rankings*
**Socioeconomic status** (income, employment, and education)	Persons below poverty[b]	Persons below poverty
	Per capita income	Per capita income
	Unemployment[b]	Persons unemployed
	No high school diploma	Persons age 25+ with no high school diploma
**Household composition and disability**	Single-parent households[b]	Single-parent households
	Persons aged 65+	Persons aged 17 and younger
	Persons aged 17 and younger	Persons aged 65+
	Population with a disability	Noninstitutionalized persons older than age 5 with a disability
**Housing type and transportation**	Multi-unit structures (10+ units)	Multi-unit housing structures (10+ units)
	Mobile homes	Mobile homes
	Crowded households[b]	Crowding (>1 person/room)
	Population w/out a vehicle[b]	Households w/ no vehicle available
	Persons in group quarters	Persons in group quarters
**Minority status and language**	Non-White	American Indian/Alaska Native, Asian, African American, Native Hawaiian/Pacific Islander, Hispanic/LatinX, other race alone
	Speak English "less than well"	Persons age 5 or older who speak English "less than well"

Abbreviations: ADI, area deprivation index; SVI, social vulnerability index.
[a] = A population-weighted mean was used to aggregate ADI block group data to tract.
[b] = Both ADI and SVI contain this item.
[C] = Negative factor loadings (lower values indicate higher deprivation).
[d] = SVI item units are percentile rankings ranging from 0 to 1.
