# Supplementary material for: Comparison of two area-level socioeconomic deprivation indices: Implications for public health research, practice, and policy
Source: PLoS One. 2023 Oct 5;18(10):e0292281. doi: 10.1371/journal.pone.0292281 (PMC10553799; doi:10.1371/journal.pone.0292281)
Supplement: S3 Table — (PDF) [file pone.0292281.s009.pdf]

Table S3. ADI and SVI Item Descriptive Statistics

**Index item**	**n** (tracts)	**Mean**	**SD**	**Min**	**Max**
**ADI 2019**[a] (*units as indicated*)	72,614	48.95	27.54	0.17	100
% Families below poverty level (0-1)[b]	73,061	0.11	0.11	0	1
Median family income $[c]	72,633	79,739.35	39212.11	2499.00	250001.00
Income disparity (ratio)	71,405	2.22	1.23	9.60	2.42
% Population <150% poverty level (0-1)	73,149	0.24	0.16	0	1
% Unemployment (0-1)[b]	73,186	0.06	0.05	0	1
% White collar occupation (0-1)[c]	73,181	0.58	0.15	0	1
% <9 years of education (0-1)	73,280	0.05	0.06	0	1
% ≥High school diploma (0-1)[c]	73,280	0.87	0.10	0	1
% Owner-occupied housing (0-1)[c]	73,113	0.63	0.23	0	1
Median monthly mortgage $[c]	71,650	1,659.25	733.88	332.00	4,001.00
Median gross rent $[c]	71,731	1,131.92	503.23	107.00	3,501.00
Median home value $[c]	71,958	271,248.80	246,013.50	9,999.00	2,000,001.00
% Single-parent households (0-1)[b]	73,061	0.15	0.11	0	1
% Households w/out vehicle (0-1)[b]	73,113	0.09	0.12	0	1
% Households w/out a telephone (0-1)	72,532	0.02	0.02	0	1
% Households, incomplete plumbing (0-1)	73,113	<0.01	0.01	0	1
% Crowded households (0-1)[b]	73,113	0.04	0.05	0	1
**SVI 2018**[d] *(percentile rankings, 0-1)*	72,173	0.50	0.29	0	1
Persons below poverty[b]	72,291	0.50	0.29	0	1
Per capita income	72,255	0.50	0.29	0	1
Unemployment[b]	72,356	0.50	0.29	0	1
No high school diploma	72,303	0.50	0.29	0	1
Single-parent households[b]	72,400	0.50	0.29	0	1
Persons aged 65+	72,411	0.50	0.29	0	1
Persons aged 17 and younger	72,411	0.50	0.29	0	1
Population with a disability	72,411	0.48	0.31	0	1
Multi-unit structures (10+ units)	72,411	0.48	0.32	0	1
Mobile homes	72,238	0.41	0.37	0	1
Crowded households[b]	72,411	0.48	0.31	0	1
Population w/out a vehicle[b]	72,228	0.50	0.29	0	1
Persons in group quarters	72,411	0.50	0.29	0	1
Minority population	72,411	0.50	0.29	0	1
Speak English “less than well”	72,411	0.42	0.36	0	1
**n, ADI and SVI (total tracts)**	**71,724**	--	--	--	--

Abbreviations: ADI, area deprivation index; SVI, social vulnerability index; %, percentage; w/out, without.
[a] = A population-weighted mean was used to aggregate ADI block group data to tract. ADI is a percentile ranking from 0 to 100.
[b] = Both ADI and SVI contain this item.
[c] = Negative factor loadings (lower values indicate higher deprivation).
[d] = All SVI item units are percentile rankings ranging from 0 to 1.
