# Supplementary material for: Comparison of two area-level socioeconomic deprivation indices: Implications for public health research, practice, and policy
Source: PLoS One. 2023 Oct 5;18(10):e0292281. doi: 10.1371/journal.pone.0292281 (PMC10553799; doi:10.1371/journal.pone.0292281)
Supplement: S4 Table — (PDF) [file pone.0292281.s010.pdf]

Table S4. Individual Index Item Mean Comparisons by Agreement: I. High ADI (10%)

**Index**<br>Item	**N**	**1b. Poor Agreement** High ADI (10%), Low SVI (40%) n	Mean	**1a. Good Agreement** High ADI (10%), High SVI (20%) n	Mean	**Difference** (1b. – 1a.)	**p-value**	**Cohen's D**
**ADI 2019** [a] (*units as indicated*)	6,771	91	90.56	4294	93.95	-3.38	**<0.001**	**0.459**
% >High school diploma[c]		91	90.04	4294	74.26	+15.78	**<0.001**	**3.203**
% Population <150% poverty level		91	30.31	4294	51.30	-20.99	**<0.001**	**2.944**
Median family income $[c]		90	53,383.03	4249	35,631.69	+17,751.35	**<0.001**	**2.737**
% <9 years of education		91	2.97	4294	10.15	-7.19	**<0.001**	**2.645**
% White collar occupation[c]		91	52.46	4294	39.39	+13.07	**<0.001**	**2.601**
% Crowded households[b]		91	0.66	4294	5.19	-4.53	**<0.001**	**2.424**
% Families below poverty level[b]		91	12.06	4294	30.31	-18.25	**<0.001**	**2.285**
% Owner-occupied housing[c]		91	69.48	4294	46.52	+22.97	**<0.001**	**2.230**
Income disparity (ratio)		90	2.86	4291	4.04	-1.18	**<0.001**	**1.756**
% Households w/out vehicle[b]		91	6.13	4294	19.51	-13.38	**<0.001**	**1.733**
% Single-parent households[b]		91	12.86	4294	29.87	-17.01	**<0.001**	**1.717**
% Unemployment[b]		91	5.06	4294	11.90	-6.84	**<0.001**	**1.194**
Median gross rent $[c]		88	711.44	4275	720.58	-9.14	0.596	**1.193**
% Households w/out a telephone		91	2.05	4202	3.57	-1.53	**<0.001**	**0.895**
Median monthly mortgage $[c]		89	937.83	4172	925.93	+11.90	0.512	0.778**
% Households, incomplete plumbing		91	0.42	4294	0.97	-0.55	0.062	0.361*
Median home value $[c]		90	67,324.44	4275	64,866.62	-2,457.83	0.208	0.253*
**SVI 2018**[d] *(percentile rankings)*	6,771	91	32.00	4294	91.11	-59.11	**<0.001**	**8.911**
No high school diploma		91	43.96	4294	84.52	-40.55	**<0.001**	**4.917**
Persons below poverty[b]		91	59.80	4294	90.43	-30.64	**<0.001**	**3.360**
Per capita income		91	61.57	4294	90.59	-29.02	**<0.001**	**3.263**
Crowded households[b]		91	15.25	4294	65.29	-50.05	**<0.001**	**2.875**
Population w/out a vehicle[b]		91	45.62	4294	81.16	-35.54	**<0.001**	**2.160**
Speak English "less than well"		91	15.28	4294	53.16	-37.88	**<0.001**	**2.159**
Minority population		91	22.73	4294	78.67	-55.94	**<0.001**	**2.135**
Single-parent households[b]		91	40.12	4294	82.92	-42.80	**<0.001**	**1.812**
Unemployment[b]		91	40.39	4294	82.24	-41.84	**<0.001**	**1.453**
Multi-unit structures (10+ units)		91	24.13	4294	49.01	-24.88	**<0.001**	**1.222**
Population with a disability		91	68.38	4294	78.35	-9.97	**<0.001**	**1.209**
Persons in group quarters		91	21.40	4294	48.93	-27.52	**<0.001**	0.721**
Persons aged 65+		91	66.13	4294	39.74	+26.40	**<0.001**	0.550**
Mobile homes		91	46.24	4294	50.11	-3.87	0.313	0.196
Persons aged 17 and younger		91	32.59	4294	73.07	-40.47	**<0.001**	0.181

Abbreviations: ADI, area deprivation index; SVI, social vulnerability index; %, percentage; w/out, without.

[a] = A population-weighted mean was used to aggregate ADI block group data to tract. ADI is a percentile ranking from 0 to 100. ADI items ranging from 0 to 1 were multiplied by 100 for comparisons.

[b] = Both ADI and SVI contain this item.

[c] = Negative factor loadings (lower values indicate higher deprivation).

[d] = SVI item units are percentile rankings ranging from 0 to 1. All SVI items were multiplied by 100 for comparisons.
Bold text: p-value, p<0.001; Cohen's D, large effect size (|Cohen's D| >0.80).

**=p-value, p<0.01; Cohen's D, medium effect size (Cohen's D | >0.50 - <0.80|).

* = p-value, p<0.05; Cohen's D, small effect size (Cohen's D |>0.20 - <0.50|).
