# Supplementary material for: Comparison of two area-level socioeconomic deprivation indices: Implications for public health research, practice, and policy
Source: PLoS One. 2023 Oct 5;18(10):e0292281. doi: 10.1371/journal.pone.0292281 (PMC10553799; doi:10.1371/journal.pone.0292281)
Supplement: S5 Table — (PDF) [file pone.0292281.s011.pdf]

Table S5. Individual Index Item Mean Comparisons by Agreement: II. High SVI (10%)

**Index** Item	**N**	**2b. Poor Agreement** High SVI (10%), Low ADI (40%)		**2a. Good Agreement** High SVI (10%), High ADI (20%)		**Difference** (2b. – 2a.)	**p-value**	**Cohen's D**
		**n**	**Mean**	**n**	**Mean**			
**ADI 2019** [a] (*units as indicated*)	7,188	1391	23.11	3655	90.49	-67.38	**<0.001**	**9.277**
Median home value $[c]		1212	426,247.28	3623	77,031.44	+349,215.84	**<0.001**	**3.924**
Median monthly mortgage $[c]		1169	2,131.59	3553	979.59	+1,152.00	**<0.001**	**3.459**
Median gross rent $[c]		1391	1,140.23	3643	736.12	+404.11	**<0.001**	**1.906**
% Crowded households[b]		1391	16.90	3655	6.57	+10.32	**<0.001**	**1.377**
% Owner-occupied housing[c]		1391	24.62	3655	44.05	-19.42	**<0.001**	**1.104**
Income disparity (ratio)		1382	3.15	3651	3.99	-0.84	**<0.001**	**0.955**
Median family income $[c]		1387	45,892.30	3624	35,989.48	+9,902.82	**<0.001**	**0.917**
% <9 years of education		1391	18.80	3655	11.93	+6.87	**<0.001**	0.780**
% Households w/out vehicle[b]		1391	32.57	3655	19.85	+12.72	**<0.001**	0.734**
% Population <150% poverty level		1391	43.06	3655	51.39	-8.33	**<0.001**	0.722**
% Families below poverty level[b]		1391	24.54	3655	30.71	-6.16	**<0.001**	0.545**
% ≥High school diploma[c]		1391	66.78	3655	72.42	-5.64	**<0.001**	0.523**
% Unemployment[b]		1391	9.43	3655	11.78	-2.36	**<0.001**	0.391*
% Single-parent households[b]		1391	26.39	3655	30.34	-3.95	**<0.001**	0.329*
% White collar occupation[c]		1391	42.16	3655	39.30	+2.86	**<0.001**	0.294*
% Households w/out a telephone		1390	2.71	3573	3.62	-0.92	**<0.001**	0.288*
% Households, incomplete plumbing		1391	0.72	3655	1.01	-0.29	**<0.001**	0.101
**SVI 2018**[d] *(percentile rankings)*	7,188	1391	94.99	3655	95.10	-0.11	0.233	0.038
Multi-unit structures (10+ units)		1391	81.57	3655	55.27	+26.30	**<0.001**	**1.095**
Population with a disability		1391	50.89	3655	75.39	-24.50	**<0.001**	**1.059**
Speak English "less than well"		1391	90.36	3655	64.59	+25.78	**<0.001**	**0.940**
Crowded households[b]		1391	91.64	3655	73.37	+18.27	**<0.001**	**0.912**
Mobile homes		1391	26.69	3655	54.22	-27.53	**<0.001**	**0.817**
Persons below poverty[b]		1391	84.60	3655	90.82	-6.22	**<0.001**	0.678**
Per capita income		1391	85.39	3655	91.06	-5.67	**<0.001**	0.634**
Minority population		1391	90.89	3655	82.28	+8.61	**<0.001**	0.620**
No high school diploma		1391	91.44	3655	86.82	+4.62	**<0.001**	0.465*
Persons aged 65+		1391	26.58	3655	37.66	-11.08	**<0.001**	0.441*
Persons aged 17 and younger		1391	71.18	3655	77.75	-6.58	**<0.001**	0.286*
Unemployment[b]		1391	77.76	3655	82.81	-5.04	**<0.001**	0.267*
Population w/out a vehicle[b]		1391	84.15	3655	81.73	+2.41	**<0.001**	0.156
Single-parent households[b]		1391	84.59	3655	85.30	-0.70	0.147	0.046
Persons in group quarters		1391	56.62	3655	56.39	+0.23	0.829	0.007

Abbreviations: ADI, area deprivation index; SVI, social vulnerability index; %, percentage; w/out, without.
[a] = A population-weighted mean was used to aggregate ADI block group data to tract. ADI is a percentile ranking from 0 to 100. ADI items ranging from 0 to 1 were multiplied by 100 for comparisons.
[b] = Both ADI and SVI contain this item.
[c] = Negative factor loadings (lower values indicate higher deprivation).
[d] = SVI item units are percentile rankings ranging from 0 to 1. All SVI items were multiplied by 100 for comparisons.
Bold text: p-value, p<0.001; Cohen's D, large effect size (|Cohen's D| ≥0.80).
**=p-value, p≤0.01; Cohen's D, medium effect size (Cohen's D | ≥0.50 - <0.80|).
* = p-value, p≤0.05; Cohen's D, small effect size (Cohen's D |≥0.20 - <0.50|).
