# Supplementary material for: Comparison of two area-level socioeconomic deprivation indices: Implications for public health research, practice, and policy
Source: PLoS One. 2023 Oct 5;18(10):e0292281. doi: 10.1371/journal.pone.0292281 (PMC10553799; doi:10.1371/journal.pone.0292281)
Supplement: S6 Table — (PDF) [file pone.0292281.s012.pdf]

Table S6. Individual Index Item Mean Comparisons by Agreement: III. Low ADI (10%)

**Index**<br>Item	**N**	**3b. Poor Agreement**<br>Low ADI (10%),<br>High SVI (40%)		**3a. Good Agreement**<br>Low ADI (10%),<br>Low SVI (20%)		**Difference**<br>(3b. – 3a.)	**p-value**	**Cohen's D**
		**n**	**Mean**	**n**	**Mean**			
**ADI 2019**[a] (*units as indicated*)	7,262	1182	7.18	3183	5.80	+1.38	**<0.001**	**0.913**
Median family income $[c]		1168	71,806.92	3178	176,633.27	-104,826.35	**<0.001**	**1.963**
% Population <150% poverty level		1182	30.45	3183	6.43	+24.03	**<0.001**	**1.900**
% Families below poverty level[b]		1182	15.16	3183	2.23	+12.93	**<0.001**	**1.586**
% ≥High school diploma[c]		1182	78.56	3183	97.27	-18.71	**<0.001**	**1.556**
Income disparity (ratio)		1163	2.42	2910	0.88	+1.54	**<0.001**	**1.510**
% White collar occupation[c]		1182	57.54	3183	83.42	-25.88	**<0.001**	**1.381**
% Single-parent households[b]		1182	14.98	3183	5.20	+9.78	**<0.001**	**1.340**
% Owner-occupied housing[c]		1182	32.94	3183	78.89	-45.95	**<0.001**	**1.301**
% Households w/out vehicle[b]		1182	34.42	3183	5.09	+29.32	**<0.001**	**1.106**
% Unemployment[b]		1182	6.45	3183	3.34	+3.11	**<0.001**	**1.038**
% <9 years of education		1182	11.77	3183	1.17	+10.60	**<0.001**	**0.918**
% Crowded households[b]		1182	12.63	3183	1.03	+11.60	**<0.001**	**0.910**
% Households w/out a telephone		1181	2.17	3169	0.89	+1.28	**<0.001**	0.453**
% Households, incomplete plumbing		1182	0.70	3183	0.20	+0.49	**<0.001**	0.198
Median home value $[c]		1090	762,927.80	3171	850,676.17	-87,748.37	**<0.001**	0.134
Median monthly mortgage $[c]		1073	2,877.21	3170	3,306.71	-429.50	**<0.001**	0.070
Median gross rent $[c]		1177	1,455.26	2906	2,173.13	-717.87	**<0.001**	0.057
**SVI 2018**[d] *(percentile rankings)*	7,262	1182	76.54	3183	8.84	+67.70	**<0.001**	**10.309**
Per capita income		1182	54.97	3183	5.59	+49.38	**<0.001**	**3.584**
Persons below poverty[b]		1182	66.93	3183	13.54	+53.39	**<0.001**	**3.494**
No high school diploma		1182	76.18	3183	10.72	+65.46	**<0.001**	**3.371**
Minority population		1182	79.01	3183	39.90	+39.11	**<0.001**	**2.693**
Single-parent households[b]		1182	53.64	3183	18.36	+35.28	**<0.001**	**2.405**
Population w/out a vehicle[b]		1182	82.06	3183	27.34	+54.72	**<0.001**	**2.269**
Unemployment[b]		1182	59.35	3183	27.97	+31.38	**<0.001**	**2.053**
Crowded households[b]		1182	87.24	3183	23.56	+63.68	**<0.001**	**1.897**
Persons aged 17 and younger		1182	42.40	3183	47.84	-5.44	**<0.001**	**1.627**
Speak English "less than well"		1182	86.37	3183	41.09	+45.28	**<0.001**	**1.108**
Persons aged 65+		1182	39.38	3183	55.01	-15.62	**<0.001**	**1.003**
Multi-unit structures (10+ units)		1182	77.68	3183	39.72	+37.95	**<0.001**	**0.916**
Persons in group quarters		1182	53.42	3183	29.02	+24.40	**<0.001**	0.754**
Population with a disability		1182	38.17	3183	15.54	+22.63	**<0.001**	0.480*
Mobile homes		1182	16.64	3183	11.91	+4.73	**<0.001**	0.107

Abbreviations: ADI, area deprivation index; SVI, social vulnerability index; %, percentage; w/out, without.
[a] = A population-weighted mean was used to aggregate ADI block group data to tract. ADI is a percentile ranking from 0 to 100. ADI items ranging from 0 to 1 were multiplied by 100 for comparisons.
[b] = Both ADI and SVI contain this item.
[c] = Negative factor loadings (lower values indicate higher deprivation).
[d] = SVI item units are percentile rankings ranging from 0 to 1. All SVI items were multiplied by 100 for comparisons.
Bold text: p-value, p<0.001; Cohen's D, large effect size (|Cohen's D| ≥0.80).
**=p-value, p≤0.01; Cohen's D, medium effect size (Cohen's D | ≥0.50 - <0.80|).
* = p-value, p≤0.05; Cohen's D, small effect size (Cohen's D |≥0.20 - <0.50|).
