# Supplementary material for: Comparison of two area-level socioeconomic deprivation indices: Implications for public health research, practice, and policy
Source: PLoS One. 2023 Oct 5;18(10):e0292281. doi: 10.1371/journal.pone.0292281 (PMC10553799; doi:10.1371/journal.pone.0292281)
Supplement: S7 Table — (PDF) [file pone.0292281.s013.pdf]

Table S7. Individual Index Item Mean Comparisons by Agreement: IV. Low SVI (10%)

**Index**<br>Item	N	4b. Poor Agreement<br>Low SVI (10%),<br>High ADI (40%)		4a. Good Agreement<br>Low SVI (10%),<br>Low ADI (20%)		Difference<br>(4b. – 4b.)	p-value	Cohen's D
		**n**	**Mean**	**n**	**Mean**			
**ADI 2019** [a] (*units as indicated*)	7,160	172	65.93	3605	10.67	-55.26	**<0.001**	**9.474**
Median monthly mortgage $[c]		169	1,162.95	3599	2,889.09	-1,726.14	**<0.001**	**2.742**
% White collar occupation[c]		172	60.99	3605	81.66	-20.67	**<0.001**	**2.550**
% Population <150% poverty level		172	13.66	3605	5.71	+7.95	**<0.001**	**2.297**
Median family income $[c]		171	76,485.78	3602	162,975.42	-86,489.64	**<0.001**	**2.268**
Median gross rent $[c]		169	873.97	3194	1,947.28	-1,073.31	**<0.001**	**1.719**
% ≥High school diploma[c]		172	94.61	3605	97.50	-2.90	**<0.001**	**1.538**
Median home value $[c]		169	127,677.51	3599	632,912.36	-505,234.85	**<0.001**	**1.506**
% Families below poverty level[b]		172	4.70	3605	2.02	+2.68	**<0.001**	**1.378**
% Single-parent households[b]		172	8.56	3605	5.03	+3.53	**<0.001**	**1.039**
Income disparity (ratio)		166	1.55	3218	0.77	+0.78	**<0.001**	**1.035**
% Households w/out a telephone		172	1.43	3559	0.87	+0.56	**<0.001**	0.525**
% <9 years of education		172	1.52	3605	0.96	+0.56	**<0.001**	0.522**
% Owner-occupied housing[c]		172	78.59	3605	84.56	-5.97	**<0.001**	0.376*
% Unemployment[b]		172	2.78	3605	3.11	-0.32	0.012*	0.196
% Crowded households[b]		172	0.43	3605	0.59	-0.15	0.036*	0.164
% Households, incomplete plumbing		172	0.18	3605	0.19	+0.00	0.949	0.005
% Households w/out vehicle[b]		172	2.73	3605	2.70	+0.02	0.953	0.005
**SVI 2018**[d] *(percentile rankings)*	7,160	172	6.72	3605	4.60	+2.12	**<0.001**	**0.732**
Per capita income		172	36.13	3605	7.27	+28.86	**<0.001**	**4.026**
Population with a disability		172	43.90	3605	15.43	+28.47	**<0.001**	**1.850**
No high school diploma		172	25.19	3605	9.46	+15.73	**<0.001**	**1.685**
Persons below poverty[b]		172	25.83	3605	10.89	+14.94	**<0.001**	**1.345**
Minority population		172	16.40	3605	32.43	-16.02	**<0.001**	**0.931**
Speak English “less than well”		172	13.90	3605	32.43	-18.53	**<0.001**	**0.853**
Mobile homes		172	31.14	3605	12.65	+18.49	**<0.001**	0.761**
Persons aged 17 and younger		172	31.30	3605	52.49	-21.19	**<0.001**	0.724**
Single-parent households[b]		172	27.28	3605	18.82	+8.46	**<0.001**	0.544**
Persons in group quarters		172	5.43	3605	20.12	-14.69	**<0.001**	0.512**
Persons aged 65+		172	64.96	3605	52.17	+12.78	**<0.001**	0.445*
Multi-unit structures (10+ units)		172	17.35	3605	30.28	-12.93	**<0.001**	0.418*
Unemployment[b]		172	20.45	3605	24.85	-4.40	0.002**	0.237*
Population w/out a vehicle[b]		172	24.16	3605	19.66	+4.50	0.005**	0.221*
Crowded households[b]		172	11.78	3605	15.54	-3.77	0.013*	0.195

Abbreviations: ADI, area deprivation index; SVI, social vulnerability index; %, percentage; w/out, without.
[a] = A population-weighted mean was used to aggregate ADI block group data to tract. ADI is a percentile ranking from 0 to 100. ADI items ranging from 0 to 1 were multiplied by 100 for comparisons.
[b] = Both ADI and SVI contain this item.
[c] = Negative factor loadings (lower values indicate higher deprivation).
[d] = SVI item units are percentile rankings ranging from 0 to 1. All SVI items were multiplied by 100 for comparisons.
Bold text: p-value, p<0.001; Cohen’s D, large effect size (|Cohen’s D| ≥0.80).
**=p-value, p≤0.01; Cohen’s D, medium effect size (Cohen’s D | ≥0.50 - <0.80|).
* = p-value, p≤0.05; Cohen’s D, small effect size (Cohen’s D |≥0.20 - <0.50|).
