# Supplementary material for: Comparison of two area-level socioeconomic deprivation indices: Implications for public health research, practice, and policy
Source: PLoS One. 2023 Oct 5;18(10):e0292281. doi: 10.1371/journal.pone.0292281 (PMC10553799; doi:10.1371/journal.pone.0292281)
Supplement: S1 File — (PDF) [file pone.0292281.s014.pdf]

Supporting Information References

1. Center for Health Disparities Research. Neighborhood Atlas: University of Wisconsin School of Medicine and Public Health; 2022 [December 15, 2022]. Available from: https://www.neighborhoodatlas.medicine.wisc.edu.
2. Agency for Toxic Substances and Disease Registry, Centers for Disease Control and Prevention. CDC SVI 2018 Documentation 2022 [December 15, 2022]. Available from: https://www.atsdr.cdc.gov/placeandhealth/svi.
3. Srivastava T, Schmidt H, Sadecki E, Kornides ML. Disadvantage indices deployed to promote equitable allocation of COVID-19 vaccines in the US: A scoping review of differences and similarities in design. JAMA Health Forum. 2022;3(1):e214501-e. doi: jamahealthforum.2021.4501.
4. Singh GK. Area deprivation and widening inequalities in US mortality, 1969–1998. American Journal of Public Health. 2003;93(7):1137-43. doi: 10.2105/AJPH.93.7.1137.
5. Kind AJ, Jencks S, Brock J, Yu M, Bartels C, Ehlenbach W, et al. Neighborhood socioeconomic disadvantage and 30 day rehospitalizations: An analysis of Medicare data. Annals of Internal Medicine. 2014;161(11):765. doi: 10.7326/M13-2946.
6. Park C, Schappe T, Peskoe S, Mohottige D, Chan NW, Bhavsar NA, et al. A comparison of deprivation indices and application to transplant populations. American Journal of Transplantation. 2023. doi: 10.1016/j.ajt.2022.11.018.
